# Association of Body Mass Index and Waist Circumference With Imaging Metrics of Brain Integrity and Functional Connectivity in Children Aged 9 to 10 Years in the US, 2016-2018

**DOI:** 10.1001/jamanetworkopen.2023.14193

**Published:** 2023-05-18

**Authors:** Simone Kaltenhauser, Clara F. Weber, Huang Lin, Ali Mozayan, Ajay Malhotra, R. Todd Constable, Julián N. Acosta, Guido J. Falcone, Sarah N. Taylor, Laura R. Ment, Kevin N. Sheth, Seyedmehdi Payabvash

**Affiliations:** 1 Department of Radiology and Biomedical Imaging, Yale School of Medicine, New Haven, Connecticut; 2 University of Regensburg, Regensburg, Germany; 3 Department of Neurology, Yale School of Medicine, New Haven, Connecticut; 4 Department of Pediatrics, Yale School of Medicine, New Haven, Connecticut

## Abstract

**Importance:**

Aside from widely known cardiovascular implications, higher weight in children may have negative associations with brain microstructure and neurodevelopment.

**Objective:**

To evaluate the association of body mass index (BMI) and waist circumference with imaging metrics that approximate brain health.

**Design, Setting, and Participants:**

This cross-sectional study used data from the Adolescent Brain Cognitive Development (ABCD) study to examine the association of BMI and waist circumference with multimodal neuroimaging metrics of brain health in cross-sectional and longitudinal analyses over 2 years. From 2016 to 2018, the multicenter ABCD study recruited more than 11 000 demographically representative children aged 9 to 10 years in the US. Children without any history of neurodevelopmental or psychiatric disorders were included in this study, and a subsample of children who completed 2-year follow-up (34%) was included for longitudinal analysis.

**Exposures:**

Children’s weight, height, waist circumference, age, sex, race and ethnicity, socioeconomic status, handedness, puberty status, and magnetic resonance imaging scanner device were retrieved and included in the analysis.

**Main Outcomes and Measures:**

Association of preadolescents’ BMI *z* scores and waist circumference with neuroimaging indicators of brain health: cortical morphometry, resting-state functional connectivity, and white matter microstructure and cytostructure.

**Results:**

A total of 4576 children (2208 [48.3%] female) at a mean (SD) age of 10.0 years (7.6 months) were included in the baseline cross-sectional analysis. There were 609 (13.3%) Black, 925 (20.2%) Hispanic, and 2565 (56.1%) White participants. Of those, 1567 had complete 2-year clinical and imaging information at a mean (SD) age of 12.0 years (7.7 months). In cross-sectional analyses at both time points, higher BMI and waist circumference were associated with lower microstructural integrity and neurite density, most pronounced in the corpus callosum (fractional anisotropy for BMI and waist circumference at baseline and second year: *P* < .001; neurite density for BMI at baseline: *P* < .001; neurite density for waist circumference at baseline: *P* = .09; neurite density for BMI at second year: *P* = .002; neurite density for waist circumference at second year: *P* = .05), reduced functional connectivity in reward- and control-related networks (eg, within the salience network for BMI and waist circumference at baseline and second year: *P* < .002), and thinner brain cortex (eg, for the right rostral middle frontal for BMI and waist circumference at baseline and second year: *P* < .001). In longitudinal analysis, higher baseline BMI was most strongly associated with decelerated interval development of the prefrontal cortex (left rostral middle frontal: *P* = .003) and microstructure and cytostructure of the corpus callosum (fractional anisotropy: *P* = .01; neurite density: *P* = .02).

**Conclusions and Relevance:**

In this cross-sectional study, higher BMI and waist circumference among children aged 9 to 10 years were associated with imaging metrics of poorer brain structure and connectivity as well as hindered interval development. Future follow-up data from the ABCD study can reveal long-term neurocognitive implications of excess childhood weight. Imaging metrics that had the strongest association with BMI and waist circumference in this population-level analysis may serve as target biomarkers of brain integrity in future treatment trials of childhood obesity.

## Key Points

**Question:**

Are body mass index (BMI) *z* scores and waist circumference associated with brain integrity and connectivity among preadolescent children?

**Findings:**

In this cross-sectional study of 4576 children, higher BMI and waist circumference were associated with reduced white matter (WM) integrity and neuronal density, lower cortical thickness, and decreased functional connectivity of cognitive control– and reward-related networks. Over a 2-year follow-up period, higher baseline BMI *z* scores were associated with lower interval development of several commissural, projection, and association WM tracts as well as the prefrontal cortex.

**Meaning:**

In this study, higher childhood BMI and waist circumference were associated with poorer imaging metrics of brain integrity and hindered interval evolution of WM microstructure, WM cytostructure, and cortex morphology.

## Teaser

This cross-sectional study evaluates the association of body mass index and waist circumference with imaging metrics that approximate brain health among participants from the Adolescent Brain Cognitive Development Study.

## Introduction

With every fifth child aged 6 to 11 years in the US being obese,^1^ childhood obesity is a growing health concern.^2^ While the concept of brain health is not universally agreed upon yet, it broadly refers to optimal brain integrity and function.^3,4^ The US Centers for Disease Control and Prevention defined brain health as an ability to “perform all the mental processes… of cognition, including the ability to learn and judge, use language, and remember.”^5^ Quantitative multimodal neuroimaging can provide objective tools for indirect assessment of different aspects of brain health.^3^ The association of children’s weight with imaging indicators of brain health remains equivocal.^6,7,8,9,10,11,12,13^ Overweight and obesity among children aged 6 to 16 years are associated with lower cognitive function,^14^ and some studies indicate that lower prefrontal cortex thickness may mediate the association of higher body mass index (BMI; calculated as weight in kilograms divided by height in meters squared) with worse executive function in children aged 9 to 11 years.^9,10^ Due to the large number of potential confounders, population-level analysis in demographically diverse cohorts are best suited to delineate the associations of higher weight with imaging metrics of brain health. The Adolescent Brain Cognitive Development (ABCD) study, as “the largest long-term study of brain development and child health in the United States,”^15^ can provide such opportunity by collecting information from more than 11 000 children enrolled in 21 centers across the US, reflecting the racial and ethnic and sociodemographic compositions of the population.^16,17^ Using baseline and 2-year follow-up information of the ABCD study, we cross-sectionally examined the association of BMI and waist circumference with multimodal magnetic resonance imaging (MRI) features of brain health. Then, we assessed the association of baseline BMI with longitudinal changes of MRI metrics over the 2-year follow-up and the association of baseline neuroimaging features with longitudinal BMI trajectories. We specifically analyzed structural MRI, resting-state functional MRI (rs-fMRI), diffusion tensor imaging (DTI), and restriction spectrum imaging (RSI) as markers of brain morphology, functional connectivity, and white matter (WM) microstructure and cytostructure, respectively.

## Methods

### Database Characteristics

We retrospectively assessed clinical and neuroimaging information of 11 878 enrollees in the ABCD study (release 4.0) at the baseline and 2-year follow-up visits. Institutional review boards (IRBs) at each study site approved the research protocol, with centralized IRB approval from the University of California, San Diego. Informed consent (parent) and assent (child) were obtained.^18^ This study followed the Strengthening the Reporting of Observational Studies in Epidemiology (STROBE) reporting guidelines for cross-sectional studies. From September 1, 2016, to August 31, 2018, 21 research centers recruited children aged 9 to 10 years through local education systems. Eligibility criteria included the absence of severe intellectual, sensory, medical, and neurological conditions.^17,19^ Questionnaires and extensive testing batteries are repeated annually, and brain imaging is obtained on the same day in 2-year intervals.^16,17,19^

### Participant Ascertainment

**Figure 1.**
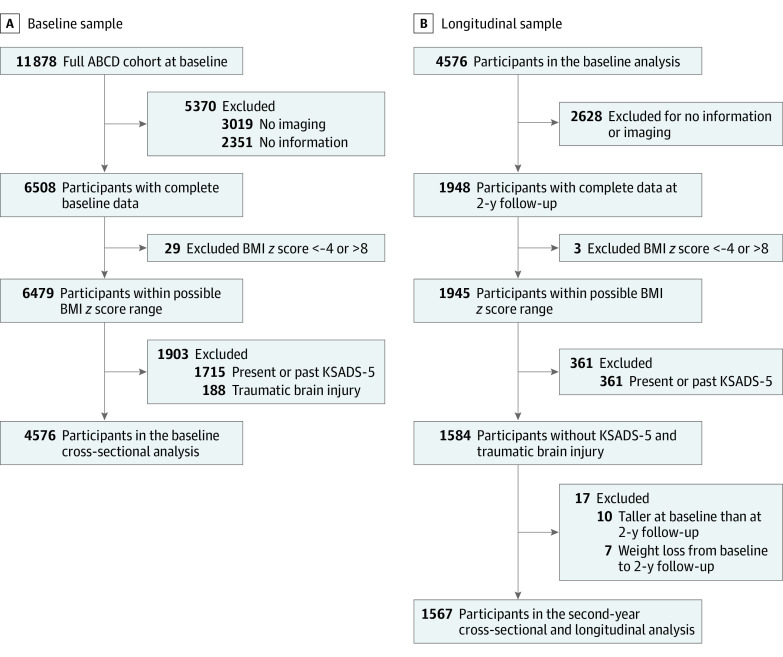
Study Flowchart ABCD indicates Adolescent Brain Cognitive Development; BMI, body mass index; and KSADS-5, Kiddie Schedule for Affective Disorders and Schizophrenia for the *DSM-5*.

We identified ABCD study participants with complete clinical and neuroimaging data at their baseline visit (Figure 1A). We further excluded children with implausible BMI *z* scores (<−4 or >8),^20^ any history of traumatic brain injury, and neurodevelopmental, psychiatric, or eating disorders as per the Kiddie Schedule for Affective Disorders and Schizophrenia for the *DSM-5* (KSADS-5).^21^ For the follow-up cross-sectional and longitudinal analyses, we identified a subsample of the baseline cohort with complete 2-year follow-up imaging and clinical information (Figure 1B) but omitted children with any weight loss and incorrect height measurements (ie, children taller at baseline than follow-up).

### Anthropometric Measurements

Anthropometric measures included waist circumference, weight *z* score, and extended BMI *z* score and percentile using the growthcleanr package in R.^22^ These values are based on the CDC age- and sex-specific reference values.^20,23^

### Sociodemographic Information

We summarized race and ethnicity reported by the parent or caregiver in the ABCD database into 6 categories: Asian, Black, Hispanic, White, multiracial or other (Alaska Native, American Indian, Guamanian, Native American, Native Hawaiian, other Pacific Islander, Samoan, other race), and no answer. As a proxy for socioeconomic status, we used the highest education of either parent or caregiver and household income (Table; eTable 1 in Supplement 1). We included handedness^25^ and calculated an overall pubertal development score according to 5 items (growth spurt in height, body hair, skin changes, facial hair growth, voice change for boys; growth spurt in height, body hair, skin changes, breast growth, and menarche for girls) allowing no more than 1 missing item.^24^

**Table.** Cohort Characteristics Across Body Mass Index Categories at Baseline

Characteristic	Participants, No. (%)					*P* value
	Total (N = 4576)	Underweight (n = 191 [4.2%])^a^	Healthy weight (n = 3046 [66.7%])^a^	Overweight (n = 683 [14.9%])^a^	Obese (n = 656 [14.3%])^a^	
Age, mean (SD), mo	119.8 (7.6)	120.8 (7.5)	119.7 (7.6)	119.8 (7.6)	119.8 (7.5)	.29
Weight, mean (SD), kg	37.1 (10.1)	26.3 (2.7)	32.8 (5.0)	42.9 (5.7)	54.5 (10.3)	<.001
Height, mean (SD), cm	140.8 (7.9)	138.9 (6.5)	139.6 (7.2)	142.9 (7.8)	144.5 (9.8)	<.001
Waist circumference, mean (SD), cm	66.8 (10.1)	57.4 (5.1)	62.5 (5.9)	72.8 (6.5)	83.2 (9.3)	<.001
BMI *z* score, mean (SD)	0.3 (1.1)	−2.2 (0.5)	−0.1 (0.7)	1.3 (0.2)	2.1 (0.5)	<.001
BMI percentile, mean (SD)	58.9 (30.7)	2.1 (1.5)	47.2 (23.0)	90.5 (2.9)	97.3 (1.5)	<.001
Puberty score^b^	1.5 (0.5)	1.4 (0.4)	1.5 (0.5)	1.6 (0.5)	1.7 (0.5)	<.001
Sex						
Male	2368 (51.7)	72 (37.7)	1708 (56.1)	320 (46.9)	268 (40.9)	<.001
Female	2208 (48.3)	119 (62.3)	1338 (43.9)	363 (54.1)	388 (59.1)	
Race and ethnicity						
Asian	97 (2.1)	9 (4.7)	71 (2.3)	5 (0.7)	12 (1.8)	<.001
Black	609 (13.3)	15 (7.9)	296 (9.7)	124 (18.2)	174 (26.5)	
Hispanic	925 (20.2)	15 (7.9)	525 (17.2)	185 (27.1)	200 (30.5)	
White	2565 (56.1)	135 (70.7)	1904 (62.5)	317 (46.4)	209 (31.9)	
Multiracial or other^c^	366 (8.0)	17 (8.9)	246 (8.1)	49 (7.2)	54 (8.2)	
No answer	14 (0.3)	0	4 (0.1)	3 (0.4)	7 (1.1)	
Parental education						
<High school	257 (5.6)	8 (4.2)	114 (3.7)	56 (8.2)	79 (12.0)	<.001
High school or GED	352 (7.7)	8 (4.2)	191 (6.3)	60 (8.8)	93 (14.2)	
Some college^d^	1081 (23.6)	32 (16.8)	621 (20.4)	201 (29.4)	227 (34.6)	
Bachelor’s degree	1195 (26.1)	66 (34.6)	856 (28.1)	151 (22.1)	122 (18.6)	
Postgraduate	1685 (36.8)	77 (40.3)	1260 (41.4)	214 (31.3)	134 (20.4)	
No answer	6 (0.1)	0 (0.0)	4 (0.1)	1 (0.1)	1 (0.2)	
Handedness						
Right	3741 (81.8)	158 (82.7)	2496 (81.9)	567 (83.0)	520 (79.3)	.60
Left	317 (6.9)	14 (7.3)	210 (6.9)	40 (5.9)	53 (8.1)	
Mixed	518 (11.3)	19 (9.9)	340 (11.2)	76 (11.1)	83 (12.7)	
Family income^e^						
<$5000	132 (2.9)	2 (1.0)	65 (2.1)	28 (4.1)	37 (5.6)	<.001
$5000-$11 999	126 (2.8)	2 (1.0)	61 (2.0)	24 (3.5)	39 (5.9)	
$12 000-$15 999	96 (2.1)	4 (2.1)	50 (1.6)	17 (2.5)	25 (3.8)	
$16 000-$24 999	164 (3.6)	2 (1.0)	83 (2.7)	31 (4.5)	48 (7.3)	
$25 000-$34 999	240 (5.2)	8 (4.2)	128 (4.2)	42 (6.1)	62 (9.5)	
$35 000-$49 999	329 (7.2)	12 (6.3)	188 (6.2)	67 (9.8)	62 (9.5)	
$50 000-$74 999	577 (12.6)	24 (12.6)	394 (12.9)	79 (11.6)	80 (12.2)	
$75 000-$99 999	641 (14.0)	27 (14.1)	438 (14.4)	96 (14.1)	80 (12.2)	
$100 000-$199 999	1395 (30.4)	73 (38.2)	1014 (33.3)	176 (25.8)	132 (20.1)	
≥$200 000	531 (11.6)	28 (14.7)	413 (13.6)	63 (9.2)	27 (4.1)	
No answer	345 (7.5)	9 (4.7)	212 (7.0)	60 (8.8)	64 (9.8)	

^a^ BMI percentile cutoffs for the BMI categories: less than 5th, underweight; between 5th and less than 85th, normal weight; between 85th and less than 95th, overweight; and 95th or greater, obese.^20^

^b^ Overall pubertal development score: range from 1 (no development) to 4 (completed development).^24^

^c^ Other race and ethnicity included Alaska Native, American Indian, Guamanian, Native American, Native Hawaiian, other Pacific Islander, Samoan, and other race.

^d^ Some college or associate degree.

^e^ Total combined family income for the past 12 months. This includes income (before taxes and deductions) from all sources. If separated or divorced, average of the 2 household incomes is used.

### Neuroimaging Metrics

Details of MRI acquisition protocols,^19^ image processing, and image analytics^26^ are provided in the eMethods in Supplement 1. The acquisition protocols were harmonized across three 3-Tesla scanner platforms (Siemens, General Electric, and Philips) at all sites.^27^ We used ABCD recommended imaging inclusion to exclude children with incidental findings that required clinical referral consideration as per a board certified neuroradiologist review and scans that failed manual quality control and review of FreeSurfer cortical surface reconstruction^26^ (eFigure 1 in Supplement 1). All series were corrected for distortions and motion. Structural MRI metrics were generated by cortical surface segmentation of T1-weighted images, and nonlinear registration to a surface-based atlas using FreeSurfer version 5.3.0.^26,28^ Cortical regions were labeled using Desikan-Killiany atlas^29^ and intracranial volume using ASEG atlas.^30^ From multi–b-value and multidirection diffusion images, DTI and RSI metrics were extracted, including fractional anisotropy (FA), mean diffusivity (MD), radial diffusivity (RD), axial diffusivity (AD), and neurite density (ND).^31^ Among DTI metrics, lower FA and higher MD indicate an overall reduction in WM fiber integrity, and elevated RD and AD reflect axonal demyelination and/or degeneration.^11,32,33^ In RSI, a tissue-based multicompartment model is applied to calculate ND, which indeed correlates with the number of neural fibers in animal studies.^34^ Major WM tracts were segmented using AtlasTrack with exclusion of voxels that included primarily gray matter or cerebrospinal fluid.^26,35^ The rs-fMRI preprocessed time courses were sampled onto the cortical surface. To characterize the functional connectome, the correlation within and between 13 predefined networks (eg, auditory, cingulo-opercular, ventral attention) were transformed to *z* statistics and averaged to measure network correlation strength.^26,36,37^ The MRI metrics of interest were average thickness of 68 cortical parcels, intracranial volume, FA, MD, RD, AD, and ND of 35 WM tracts and 91 functional connectivity correlations. These regions of interest (ROIs) are easily replicable and freely available within the data release 4.0.

### Statistical Analysis

Discrete variables are reported as counts and percentages and continuous variables as means and SDs. We compared MRI and sociodemographic variables between BMI categories using 1-way analysis of variance, χ^2^ test, and Fisher exact test. At baseline and follow-up, separate cross-sectional linear regression models evaluated the association of BMI *z* scores, weight *z* scores, and waist circumference (all continuous variables) with neuroimaging metrics. For the longitudinal analysis, we calculated the interval changes in MRI metrics and BMI *z* scores by subtracting the baseline from the 2-year follow-up values for each participant. We used paired *t* test to compare the means between each baseline and follow-up metric. Linear regression models were used to examine associations of baseline BMI *z* scores with interval changes in neuroimaging metrics as well as associations of baseline MRI variables with interval changes in BMI *z* scores. We also constructed BMI *z* score categories to compare demographic and imaging metrics between these groups (eMethods in Supplement 1). In addition, we compared linear vs different polynomial model fits between BMI *z* scores and imaging metrics (eMethods in Supplement 1).^20^ We adjusted each regression for children’s age, sex, race and ethnicity, socioeconomic status (ie, parental highest education and family income), handedness, puberty, intracranial volume, and MRI device serial number as well as multiple testing using the Benjamini-Hochberg method.^38^ We reported adjusted *P* values with the significance level set at .05. Statistical analyses were conducted using R statistical software version 4.2.0 (R Project for Statistical Computing). Additional R packages used for data cleaning, analysis, and visualization included car,^39^ tidyverse,^40^ dplyr,^41^ MASS,^42^ ggplot2,^43^ cowplot,^44^ ggseg,^45^ circlize.^46^

## Results

### Participants’ Demographic Characteristics

A total of 4576 children at a mean (SD) age of 10.0 years (7.6 months) were included in the baseline cross-sectional analysis (Figure 1A). There were 2208 (48.3%) female participants; 609 (13.3%) Black, 925 (20.2%) Hispanic, and 2565 (56.1%) White participants. Of those, 1567 with a mean (SD) age of 12.0 years (7.7 months) were included in the follow-up cross-sectional and longitudinal analyses (Figure 1B). As per BMI percentiles, 1339 (29.2%) and 450 (28.8%) children had overweight or obesity at baseline and 2-year follow-up, respectively (Table; eTable 1 in Supplement 1).^20^

### Association of WM Microstructure and Cytostructure With BMI and Waist Circumference

Higher BMI *z* scores and waist circumference were associated with pervasive reductions of averaged FA in both hemispheres (*P* < .001) (eFigure 2 in Supplement 1), which were most pronounced in the corpus callosum (FA for BMI and waist circumference at baseline and second year: *P* < .001), forceps major and minor (*P* < .009), fornices (*P* < .001), and superior longitudinal fasciculi (*P* < .03). Averaged MD of both hemispheres was not significantly associated with BMI *z* scores or waist circumference, but higher waist circumference was associated with decreased MD in the corpus callosum including forceps minor (*P* < .01). Higher BMI *z* scores were associated with higher RD of both hemispheres (*P* < .001), and higher waist circumference with higher RD of both inferior-fronto-occipital fasciculi and corticospinal/pyramidal tracts (*P* < .03 for all). Also, higher waist circumference was associated with lower AD in WM tracts of both hemispheres (*P* < .05), whereas the association of BMI *z* scores with AD reductions was more pronounced in the right hemisphere (*P* = .03), including the cingulate gyrus (*P* = .01) and uncinate fasciculus (*P* = .02), as well as the corpus callosum (*P* = .002) and bilateral superior longitudinal fasciculi (*P* < .002). Finally, we found pervasive reductions of averaged ND in both hemispheres with higher BMI *z* scores and waist circumference (ND for BMI at baseline: *P* < .001; ND for waist circumference at baseline: *P* = .09; ND for BMI at second year: *P* = .002; ND for waist circumference at second year: *P* = .05). In the cross-sectional analysis at the 2-year follow-up, similar patterns were found (eFigure 3 in Supplement 1).

### Association of Cortical Morphology With BMI and Waist Circumference

**Figure 2.**
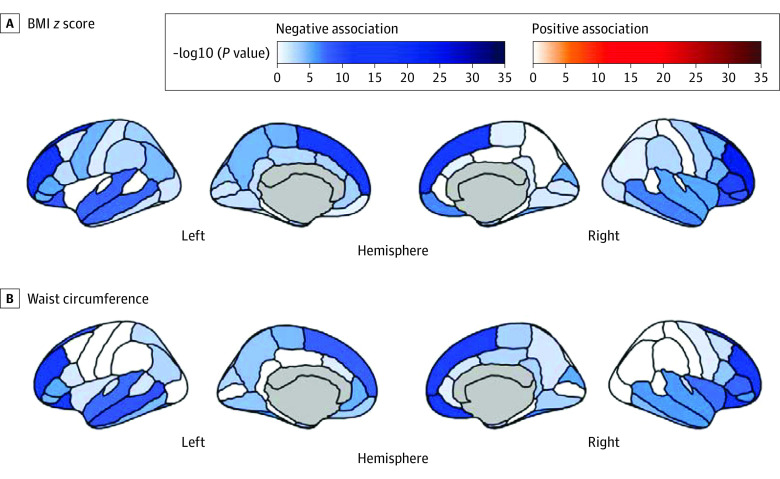
Association of Cortex Morphology With Higher Body Mass Index (BMI) *z* Scores and Waist Circumference at Baseline

Higher BMI *z* scores and waist circumference were predominantly associated with thinner brain cortex in both hemispheres (*P* < .001), including the caudal anterior cingulate gyri (*P* < .04) (Figure 2). For both BMI *z* scores and waist circumference, the strongest associations with cortical thickness were present in prefrontal cortical regions (eg, right rostral middle frontal for BMI and waist circumference at baseline and second year: *P* < .001). Similar patterns, however less extensive, were found in the 2-year follow-up cross-sectional analysis (eFigure 4 in Supplement 1).

### Association of Functional Connectivity With BMI and Waist Circumference

Higher BMI *z* scores and waist circumference at baseline had predominantly negative association with rs-fMRI brain connectivity (eFigure 5 in Supplement 1). The strongest associations were with intranetwork and between-network correlations of salience and cingulo-opercular networks^36^ (eg, within the salience network for BMI and waist circumference at baseline and second year: *P* < .002). The connectivity between the sensorimotor hand and visual network was positively associated with BMI *z* scores and waist-circumference (*P* < .04), as was the connectivity between the default and dorsal attention network with BMI *z* scores (*P* < .001). We found similar patterns in the cross-sectional analysis of the 2-year follow-up scans (eFigure 6 in Supplement 1).

### Longitudinal Changes of Neuroimaging Metrics

Over the 2-year follow-up period (mean [SD], 23.8 [1.6] months), we observed overall interval cortical thinning (eFigure 7 in Supplement 1). The averaged FA and ND of WM tracts predominantly increased, whereas MD, RD. and AD decreased (*P* < .001 for all). Intranetwork and internetwork correlations of functional connectivity changed in different directions (eFigure 7 in Supplement 1); specifically, the connectivity of ventral attention, default, and none networks predominantly decreased, whereas, the connectivity of cingulo-parietal and sensorimotor hand and mouth networks increased (*P* < .001 for all).

### Association of Baseline BMI With Longitudinal Changes of Neuroimaging Metrics

**Figure 3.**
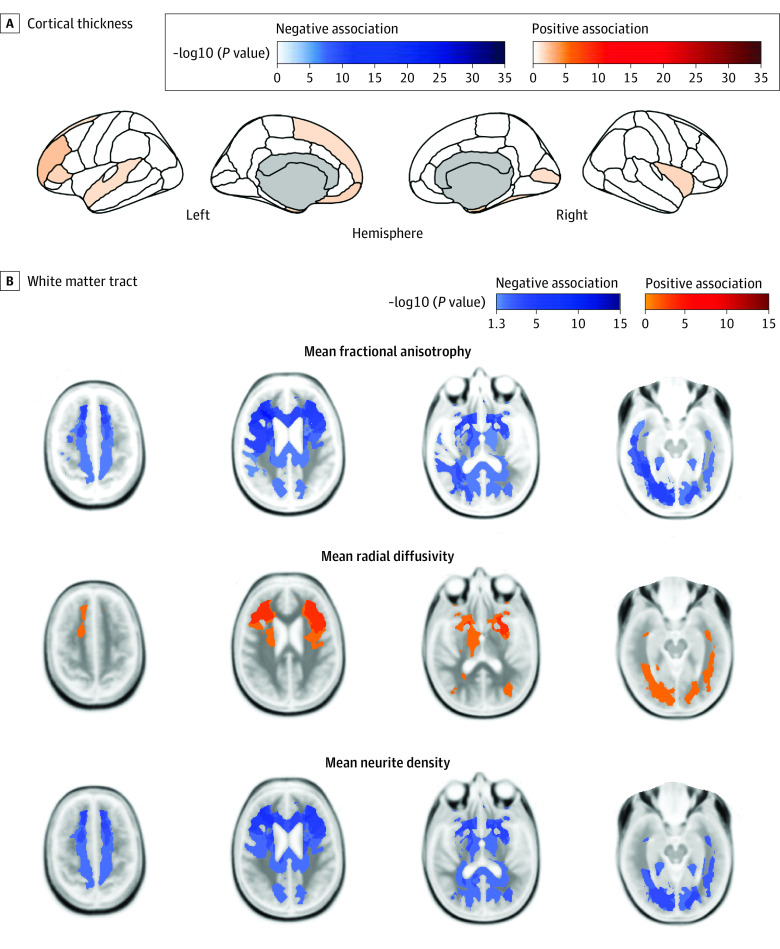
Association of Baseline Body Mass Index *z *Scores With Interval Changes in Magnetic Resonance Imaging Features

Higher baseline BMI *z* scores were negatively associated with interval changes in ND and FA of bilateral inferior-fronto-occipital fasciculi, anterior thalamic radiations, striatal inferior frontal cortices, and corpus callosum, including forceps minor, ND of the right cingulate gyrus, and FA of the right inferior longitudinal fasciculus and superior longitudinal fasciculus (Figure 3). Higher BMI *z* scores at baseline were positively associated with interval changes in RD of bilateral inferior-fronto-occipital fasciculi, left striatal inferior frontal cortex, and right anterior thalamic radiation. There were significant positive associations between baseline BMI *z* scores and interval changes in cortical thickness of 11 regions, especially in the prefrontal cortex (Figure 3). On the other hand, when analyzing whether any of the baseline MRI metrics had significant association with interval BMI *z* score changes during the 2-year follow-up, no baseline neuroimaging feature was associated with interval increase in BMI.

### Association of Weight *z* Scores With Neuroimaging Metrics

Using weight *z* scores as the explanatory variable revealed similar results as BMI *z* scores and waist circumference. eFigures 8 and 9 in Supplement 1 show results.

### Association of BMI Categories With Neuroimaging Metrics and Polynomial Modeling

The results of the linear regression were compared across BMI categories (eTable 2 and eFigures 10-13 in Supplement 1). Also, in comparison with polynomial models (up to the fifth degree), we found that the linear model can sufficiently determine the association between anthropometric measures and MRI variables (eTables 3-5 and eFigure 14 in Supplement 1).

## Discussion

Based on a large demographically representative cohort of North American preadolescents, we found that higher BMI and waist circumference were largely associated with lower WM tract microstructural and cytostructural integrity (most notably in the corpus callosum), thinner brain cortex (most pronounced in the frontal lobe), and reduced functional connectivity (especially in control- and reward-related networks) in predominantly both hemispheres. The BMI-associated neuroimaging patterns of poorer brain integrity were similar when using BMI categories instead of a continuous BMI metric and at the 2-year follow-up. In longitudinal analysis, higher baseline BMI was associated with lower interval increment in ND and FA of commissural, projection, and association WM tracts as well as interval cortical thinning of several frontal and temporal regions. While cross-sectional studies cannot establish a causal relationship, our longitudinal analysis suggests possible contribution of higher BMI to hindered interval development of WM microstructure and cytostructure and cortex morphology in preadolescents.

While some prior studies have tested specific hypotheses with focuses on select brain circuits or neurostructures in smaller cohorts, our analysis provides a comprehensive picture of which microstructural, morphological, and connectivity metrics are most strongly associated with BMI and waist circumference among preadolescents. The neuroimaging metrics with the strongest association with BMI and waist circumference may serve as target biomarkers in future clinical trials for treatment of childhood obesity. Our results also set the stage for longitudinal analysis of the association between childhood BMI and its neuroimaging correlates with long-term cognitive performance of ABCD participants in future follow-ups.

In our study, higher BMI and waist circumference were associated with pervasive reductions of FA—the primary index of WM integrity^32^—and parallel reductions of RSI-driven ND, which point to poorer microstructural integrity, in part due to lower neuronal counts.^47^ During pediatric neurodevelopment, including the age span of the participants in the present study, increasing FA and ND of WM tracts represents incremental organization of WM fiber tracts and, thus, increasing linearity of water molecule movements and neural count.^48,49^ Since baseline BMI was negatively associated with longitudinal interval changes of FA and ND within association, projection, and commissural WM tracts, a causal role of higher baseline BMI in impeding expected interval increment of FA and ND is plausible. In both cross-sectional analyses, lower AD and higher RD indicated less diffusion along, and increased diffusion perpendicular to, the axonal tracts and, thus, poor axonal and myelin integrity associated with higher BMI and waist circumference.^11,32,33^ The age-dependent decrease of MD, RD, and AD is in line with incremental organization of WM fiber tracts during childhood and adolescence.^48,49^ Thus, the positive association of higher baseline BMI with interval changes of RD further supports hindered development of WM architecture with higher BMI at baseline.

The reduced prefrontal cortical thickness in association with higher BMI and waist circumference is consistent with previous reports on smaller ABCD subsamples and pediatric cohorts.^9,10,50^ In addition, other groups reported that lower prefrontal cortex thickness mediates the association of childhood obesity with compromised executive function and working memory.^9,10^ It is speculated that BMI-associated thinner prefrontal cortex and related impaired working memory may in turn contribute to poor dietary decision-making.^9,10,51^ Given the complex pathophysiology of obesity that involves biological, psychological, social, and environmental factors, it is likely that the association between BMI and brain health imaging markers is reciprocal.^52^ Genetic variance also plays a role in these morphological differences in brain cortex and associated behaviors that are involved in food consumption.^53,54,55^

We found interval cortical thinning from children’s mean age of 10 to 12 years, as previously reported throughout adolescence.^56^ Of note, rather than absolute level of cortical thickness, the trajectory and extent of changes are associated with children’s intelligent quotient.^57,58^ Specifically, higher intellectual abilities are associated with accelerated increase in cortical thickness up to age 11.2 years, followed by accelerated decrease.^57,58^ Thus, the positive association of interval changes in cortical thickness with baseline BMI may represent deceleration in expected evolution of cortical morphology.

Finally, higher BMI and waist circumference were predominantly associated with decreased intranetwork and internetwork functional connectivity, involving the salience and cingulo-opercular networks.^36,59,60^ Both networks encompass the dorsal anterior cingulate cortex (dACC)^59,60^ which is involved in cognitive control, motivation, and reward-based decision-making.^61,62^ Additionally, thickness of bilateral dACC was reduced with higher BMI and waist circumference. Thus, our findings are consistent with another study reporting reduced functional connectivity strength within the dACC among individuals with obesity compared with those with normal weight.^63^ In conjunction with the thinning of prefrontal cortical parcels, which are important in top-down inhibitory control,^52^ the reduced functional connectivity may represent impaired regulation of reward-driven behavior among children with higher BMI and waist circumference.

### Limitations

There are several limitations to our study. First, our analysis is limited by using processed imaging metrics from the ABCD Consortium. Although we excluded children with any KSADS diagnoses, including substance use and eating disorders, we could not account for all medication uses that may have affected weight or appetite. Furthermore, the lack of physical activity as a covariable is a limitation of our study. In addition, children from lower socioeconomic status are not fully represented in our analysis. Although we adjusted for puberty levels, scanner machines, and intracranial volumes in our multivariate models, these variables can ideally be adjusted in nested analysis. The cross-sectional design and short-term longitudinal follow-up limit causal inference and definite mechanistic analysis. The exact associations of brain region and respective function with BMI and waist circumference need to be elucidated in long-term mediation and interventional trials.

## Conclusions

While cardiovascular implications of higher weight in adults and children are well established, our study highlights the association of higher BMI and waist circumference with imaging metrics of poorer brain integrity in children. As recently highlighted in the Clinical Practice Guideline for the Evaluation and Treatment of Children and Adolescents With Obesity by the American Academy of Pediatrics,^64^ our results further underscore the importance of providing effective care and treatment to children and adolescents with overweight and obesity. Although cross-sectional studies cannot establish a causal relationship, our longitudinal analysis suggests that higher childhood BMI is associated with decelerated development of WM microstructure and frontal cortex morphometry. Future results from the ongoing ABCD Study will help determine long-term cognitive consequences of higher childhood BMI. Neuroimaging patterns in association with higher BMI and waist circumference inferred from this population-level study may potentially serve as target biomarkers in future treatment trials of childhood obesity.

## Notes

Supplement 1.
eMethods.
**eTable 1.** Cohort Characteristics Across BMI Categories at the Second-Year Follow-up **eTable 2.** Comparison of Neuroimaging Metrics Between BMI Categories **eTable 3.** Comparison of the Bayesian Information Criterion Between the Linear and Polynomial Model for the Most Significant Imaging Metrics **eTable 4.** Comparison of the Bayesian Information Criterion Between the Linear and Polynomial Models for the Most Significant WM Tract Fractional Anisotropy **eTable 5.** Comparison of the Mean Squared Error (MSE) of the Linear and Different Polynomial Degree Models **eFigure 1.** Image Inclusion and Exclusion Process **eFigure 2.** Association of WM Microstructural and Cytostructural Markers With Higher BMI z Scores and Waist Circumference in the Baseline Cross-sectional Analysis **eFigure 3.** Association of WM Microstructural and Cytostructural Markers With Higher BMI z Scores and Waist Circumference in the Second-Year Follow-up Cross-sectional Analysis **eFigure 4.** Association of Brain Cortex Morphology With Higher BMI z Scores and Waist Circumference in the Second-Year Follow-up Cross-sectional Analysis **eFigure 5.** Association of Functional Connectivity With BMI z Scores and Waist Circumference in the Baseline Cross-sectional Analysis **eFigure 6.** Association of Functional Connectivity With BMI z Scores and Waist Circumference in the Second-Year Follow-up Cross-sectional Analysis **eFigure 7.** Interval Changes of Cortical Thickness, WM Microstructure and Cytostructure, and Functional Connectivity From Baseline to Second-Year Follow-up **eFigure 8.** Association of Cortical Thickness, WM Tract Microstructural and Cytostructural Markers, and Functional Connectivity With Weight z Scores in the Baseline Cross-sectional Analysis **eFigure 9.** Association of Cortical Thickness, WM Tract Microstructural and Cytostructural Markers, and Functional Connectivity With Weight z Scores in the Second-Year Follow-up Cross-sectional Analysis **eFigure 10.** Significant Differences in WM Microstructure and Cytostructure Between BMI Categories in the Baseline Cross-sectional Analysis **eFigure 11.** Significant Differences in WM Microstructure and Cytostructure Between BMI Categories in the Second-Year Follow-up Cross-sectional Analysis **eFigure 12.** Significant Differences in Brain Cortex Morphology Between BMI Categories in the Baseline and Second-Year Follow-up Cross-sectional Analyses **eFigure 13.** Significant Differences in Functional Connectivity Between the Groups With Normal Weight and Obesity in the Baseline and Second-Year Cross-sectional Analyses **eFigure 14.** Association of Average Fractional Anisotropy With BMI z Scores in the Baseline Cross-sectional Analysis
eReferences.

Supplement 2.
Data Sharing Statement
