# Supplementary material for: Association of Body Mass Index and Waist Circumference With Imaging Metrics of Brain Integrity and Functional Connectivity in Children Aged 9 to 10 Years in the US, 2016-2018
Source: JAMA Netw Open. 2023 May 18;6(5):e2314193. doi: 10.1001/jamanetworkopen.2023.14193 (PMC10196880; doi:10.1001/jamanetworkopen.2023.14193)
Supplement: Supplement 1. — eMethods. eTable 1. Cohort Characteristics Across BMI Categories at the Second-Year Follow-up eTable 2. Comparison of Neuroimaging Metrics Between BMI Categories eTable 3. Comparison of the Bayesian Information Criterion Between the Linear and Polynomial Model for the Most Significant Imaging Metrics eTable 4. Comparison of the Bayesian Information Criterion Between the Linear and Polynomial Models for the Most Significant WM Tract Fractional Anisotropy eTable 5. Comparison of the Mean Squared Error (MSE) of the Linear and Different Polynomial Degree Models eFigure 1. Image Inclusion and Exclusion Process eFigure 2. Association of WM Microstructural and Cytostructural Markers With Higher BMI z Scores and Waist Circumference in the Baseline Cross-sectional Analysis eFigure 3. Association of WM Microstructural and Cytostructural Markers With Higher BMI z Scores and Waist Circumference in the Second-Year Follow-up Cross-sectional Analysis eFigure 4. Association of Brain Cortex Morphology With Higher BMI z Scores and Waist Circumference in the Second-Year Follow-up Cross-sectional Analysis eFigure 5. Association of Functional Connectivity With BMI z Scores and Waist Circumference in the Baseline Cross-sectional Analysis eFigure 6. Association of Functional Connectivity With BMI z Scores and Waist Circumference in the Second-Year Follow-up Cross-sectional Analysis eFigure 7. Interval Changes of Cortical Thickness, WM Microstructure and Cytostructure, and Functional Connectivity From Baseline to Second-Year Follow-up eFigure 8. Association of Cortical Thickness, WM Tract Microstructural and Cytostructural Markers, and Functional Connectivity With Weight z Scores in the Baseline Cross-sectional Analysis eFigure 9. Association of Cortical Thickness, WM Tract Microstructural and Cytostructural Markers, and Functional Connectivity With Weight z Scores in the Second-Year Follow-up Cross-sectional Analysis eFigure 10. Significant Differences in WM Microstructure and Cytostructu [file jamanetwopen-e2314193-s001.pdf]

Supplemental Online Content

Kaltenhauser S, Weber CF, Lin H, et al. Association of body mass index and waist circumference with imaging metrics of brain integrity and functional connectivity in children aged 9 to 10 years in the US, 2016-2018. *JAMA Netw Open.* 2023;6(5):e2314193. doi:10.1001/jamanetworkopen.2023.14193

eMethods.

eTable 1. Cohort Characteristics Across BMI Categories at the Second-Year Follow-up

eTable 2. Comparison of Neuroimaging Metrics Between BMI Categories

eTable 3. Comparison of the Bayesian Information Criterion Between the Linear and Polynomial Model for the Most Significant Imaging Metrics

eTable 4. Comparison of the Bayesian Information Criterion Between the Linear and Polynomial Models for the Most Significant WM Tract Fractional Anisotropy

eTable 5. Comparison of the Mean Squared Error (MSE) of the Linear and Different Polynomial Degree Models

eFigure 1. Image Inclusion and Exclusion Process

eFigure 2. Association of WM Microstructural and Cytostructural Markers With Higher BMI *z* Scores and Waist Circumference in the Baseline Cross-sectional Analysis

eFigure 3. Association of WM Microstructural and Cytostructural Markers With Higher BMI *z* Scores and Waist Circumference in the Second-Year Follow-up Cross-sectional Analysis

eFigure 4. Association of Brain Cortex Morphology With Higher BMI *z* Scores and Waist Circumference in the Second-Year Follow-up Cross-sectional Analysis

eFigure 5. Association of Functional Connectivity With BMI *z* Scores and Waist Circumference in the Baseline Cross-sectional Analysis

eFigure 6. Association of Functional Connectivity With BMI *z* Scores and Waist Circumference in the Second-Year Follow-up Cross-sectional Analysis

eFigure 7. Interval Changes of Cortical Thickness, WM Microstructure and Cytostructure, and Functional Connectivity From Baseline to Second-Year Follow-up

eFigure 8. Association of Cortical Thickness, WM Tract Microstructural and Cytostructural Markers, and Functional Connectivity With Weight *z* Scores in the Baseline Cross-sectional Analysis

eFigure 9. Association of Cortical Thickness, WM Tract Microstructural and Cytostructural Markers, and Functional Connectivity With Weight *z* Scores in the Second-Year Follow-up Cross-sectional Analysis

eFigure 10. Significant Differences in WM Microstructure and Cytostructure Between BMI Categories in the Baseline Cross-sectional Analysis

eFigure 11. Significant Differences in WM Microstructure and Cytostructure Between BMI Categories in the Second-Year Follow-up Cross-sectional Analysis

eFigure 12. Significant Differences in Brain Cortex Morphology Between BMI Categories in the Baseline and Second-Year Follow-up Cross-sectional Analyses

eFigure 13. Significant Differences in Functional Connectivity Between the Groups With Normal Weight and Obesity in the Baseline and Second-Year Cross-sectional Analyses

eFigure 14. Association of Average Fractional Anisotropy With BMI *z* Scores in the Baseline Cross-sectional Analysis

© 2023 Kaltenhauser S et al. *JAMA Network Open.*

eReferences.

This supplemental material has been provided by the authors to give readers additional information about their work.

© 2023 Kaltenhauser S et al. *JAMA Network Open.*

eMethods.

Imaging acquisition and post-processing workflow

In the Adolescent Brain Cognitive Development (ABCD) study, standard modality-specific pre-processing that included conversion from raw to compressed files, correction for distortion and movement and alignment to standard space were performed for all series[1].

Structural magnetic resonance imaging (MRI) scans were corrected for gradient nonlinearity distortions[2,3]. T2-weighted images were registered to T1-weighted images using mutual information[3]. Intensity non-uniformity correction was based on tissue segmentation and sparse spatial smoothing. T1-weighted brain MRIs were resampled with 1 mm isotropic voxels into rigid alignment with standardized MNI-152 brain. Cortical surface reconstruction was performed using FreeSurfer v5.3.0 (https://surfer.nmr.mgh.harvard.edu) and included skull stripping[4], white matter segmentation and initial mesh creation[5], correction for topological defects[6,7], surface optimization[5,8,9] and nonlinear registration to a spherical surface-based atlas[10]. Parcellation and labelling with a surface-based atlas provided brain regions of interest[9,11,12]. Cortical regions were labelled using Desikan-Killiany atlas[12] and intracranial volume using ASEG atlas[11].

Multi-b-value, multi-direction diffusion weighted images were corrected for eddy current distortion with a nonlinear estimation using diffusion gradient orientations and amplitudes to predict the pattern of distortions[13] as well as head motion by registering to images synthesized from tensor fit[14]. Diffusion gradients were adjusted for head rotation[14,15]. Robust diffusion tensor estimation[16] was used to identify and replace dark slices caused by abrupt head motion. B_0 distortions were corrected using the reversing gradient method with FSL's TOPUP[17,18] followed by gradient nonlinearity distortion correction[2], registration of T2-weighted b=0 images to T1-weighted structural images using mutual information[3] and resampling into a standard orientation with 1.7 mm isotropic resolution. Using conventional diffusion tensor imaging (DTI) methods[19-21], microstructural tissue properties, fractional anisotropy (FA), mean (MD), radial (RD) and axial diffusivity (AD) were measured. Linear estimation approach allowed for mixtures of "restricted" and "hindered" diffusion pools within individual voxels[22-24]. Two volume fractions were modeled as fiber orientation density (FOD) functions: longitudinal diffusivity constant for both fractions, with a value of $1x10^{-3}$ mm^2/s; restricted fraction (e.g. intracellular): transverse diffusivity modelled as 0; hindered fraction (e.g. extracellular): transverse diffusivity modelled as 0.9 x $1x10^{-3}$ mm^2/s. Primary measures derived from the restriction spectrum imaging (RSI) model fit included restricted normalized directional or "neurite density" (ND): sum of squared 2nd and 4th order spherical harmonic coefficients of the restricted fraction divided by the norm of the model coefficients. Major white matter (WM) tracts were labelled using AtlasTrack[1,14] with exclusion of voxels containing primarily gray matter or cerebral spinal fluid.

Resting state functional MRIs (rs-fMRI) were generated and processed using head motion correction by registering each frame to the first using AFNI's 3dvolreg[25]. B_0 distortions were corrected using the reversing gradient method with FSL's TOPUP[17,18]. The displacement field was estimated from spin-echo field map scans, followed by correction for gradient nonlinearity distortions[2], between scan motion correction across all fMRI scans in imaging event and registration between T2-weighted, spin-echo B0 calibration scans and T1-weighted structural images using mutual information[3]. Rs-fMRI specific pre-processing further included removal of initial volumes (Siemens: 8 TRs, Philips: 8 TRs, GE DV25: 5 TRs, GE DV26: 16 TRs), normalization and demean (divide by the mean of each voxel, subtract 1, multiply by 100), linear regression to remove quadratic trends and signals correlated with motion and mean time courses of cerebral WM, ventricles, and whole brain, plus first derivatives[26,27]. The motion regression included 6 parameters plus derivatives and squares with exclusion of frames with displacement >0.3 mm from the regression[26]. Consequently, the band-pass was filtered between 0.009 and 0.08 Hertz[28]. The pre-processed time courses were sampled onto the cortical surface. Head and respiratory motions were censored as described before[26,29]. Parcellation and labelling was performed according to parcels from resting-state functional connectivity patterns[30]. Functional connectivity analysis was conducted in a seed-based correlational approach[31]. Average within- and between-network mean correlations were calculated between each pairs of regions of interest (ROIs), Fisher transformed to z-statistics and averaged to provide a measure of network correlation strength[31].

Comparison of MRI metrics between Body Mass Index (BMI) categories

In addition to linear regression analysis, we categorized children into four BMI-z-score groups: < 5th underweight, ≥ 5th and < 85th normal weight, ≥ 85th and < 95th overweight, and ≥ 95th obese at baseline and second-year follow-up (Table 1, eTables 1 and 2)[32]. Then, we compared BMI category pairs with each other using the group with the lower BMI as reference, respectively. Similar to the linear regression models, analyses were corrected for age, sex, race/ethnicity, handedness, socioeconomic status, MRI scanner, puberty and intracranial volume.

Exploration of linear versus polynomial association between MRI metrics and BMI

To characterize the relationship between anthropometric measures and MRI metrics, we compared unadjusted linear models to polynomial models. We selected the best polynomial model by fitting a polynomial relationship

© 2023 Kaltenhauser S et al. *JAMA Network Open.*

between MRI metric and BMI-z-scores as the explanatory variable including up to the 5th degree of the polynomial correlation and using backwards variable selection on the degrees. We compared the Bayesian information criterion (BIC)[33] between the polynomial and linear model using WM tract, cortical region and Gordon functional connectivity correlation where the relationship of the respective MRI metric with BMI-z-scores was most significant in the cross-sectional analysis at baseline. As an example, we plotted the best polynomial model for the five WM tracts that had the lowest p-value in the cross-sectional analysis of the association between FA and BMI percentile at baseline. Furthermore, we compared the mean squared error (MSE) of five different polynomial degree regression models with the linear model. To do so, we used 10-fold cross-validation to calculate the MSE for each polynomial model and each WM tract, cortical region and Gordon functional connectivity correlation, respectively. To account for every ROI, where the respective MRI metric was measured, we calculated an average MSE for every polynomial model of each MRI metric by dividing the sum of MSEs of all ROIs by the number of ROIs of each MRI metric. For every MRI metric separately, we used one-way ANOVA test to compare whether these average MSEs of the different polynomial models significantly differ from each other.

eResults.

Relationship of neuroimaging metrics with BMI-z-scores and waist-circumference in the second-year follow-up cross-sectional analysis

Relationship of WM microstructure and cytostructure with BMI and waist-circumference
The eFigure 3 summarizes the association of WM tract FA, MD, RD, AD and ND with higher BMI-z-scores and waist-circumference in the second-year follow-up cross-sectional analysis. Overall, the results are on par with those from the baseline cross-sectional analysis (Fig. 2). Higher BMI-z-scores and waist-circumference were associated with reduced averaged FA in both hemispheres (P<.001) across many WM tracts – most prominently in the corpus callosum, inferior-fronto-occipital fasciculi and fornices (P<.001 for all). Averaged MD was not significantly changed on the hemispheric level with higher BMI-z-scores or waist-circumference, however, we found lower MD in the left cingulum (P=.03), corpus callosum (P=.05) and forceps minor (P=.05) in relation to higher waist-circumference. With higher BMI-z-scores, RD was elevated in both hemispheres (P=.008), foremost in both inferior-fronto-occipital fasciculi (P=.006), corticospinal/pyramidal tracts (P<.04) and corpus callosum (P=.03). Waist-circumference had no significant association with RD of whole hemispheres or specific WM tracts. Higher BMI-z-scores and waist-circumference were associated with decrease in averaged AD of WM tracts of both hemispheres (P<.001), with the strongest associations in the corpus callosum including the forceps minor and major, both uncinate fasciculi and cingulate gyri (P<.001 for all). Increased BMI-z-scores were associated with ND reductions of both hemispheres (P=.001) – most pronounced in the inferior-fronto-occipital fasciculi (P=.001), corpus callosum (P=.002) and superior longitudinal fasciculi (P<.004). With higher waist-circumference, we found lower ND only in the right hemisphere (P=.04), more specifically in the right (P=.009) and left inferior-fronto-occipital fasciculus (P=.02), right fornix (P=.04) and right uncinate fasciculus (P=.04).

Relationship of cortical morphology with BMI-z-scores and waist-circumference
The eFigure 4 summarizes the associations of cortical thickness with higher BMI-z-scores and waist-circumference at the second-year follow-up. Higher BMI-z-scores and waist-circumference were predominantly associated with thinner cortex in the right hemisphere (P≤.03). Similar to the baseline cross-sectional analysis (Fig. 3), the most prominent associations of cortical thickness with BMI-z-scores included the right rostral middle frontal (P<.001) and right lateral orbitofrontal regions (P=.03). With respect to waist-circumference, the most significant correlations with thickness were found in the right rostral middle frontal (P<.001), right lateral orbitofrontal (P=.008) and right pars triangularis (P=.008).

Relationship of functional connectivity with BMI-z-scores and waist-circumference
On par with the baseline cross-sectional analysis (eFigure 5), higher BMI-z scores and waist-circumference had predominantly negative association with Gordon network correlations (eFigure 6). Except for one positive association of BMI-z-scores with the correlation between the default and dorsal attention network (P=.003), all significant associations were negative. With respect to both BMI-z-scores and waist-circumference, the most pronounced functional connectivity reductions included the correlation within the salience network (P<.001 for both), within the cingulo-opercular network (P<.001 for BMI-z-scores and P=.02 for waist-circumference) as well as the correlation between the salience and cingulo-opercular network (P=.04 for BMI-z-scores).

Relationship of neuroimaging metrics with weight-z-scores in the baseline and second-year follow-up cross-sectional analyses

Relationship of WM microstructure and cytostructure with weight-z-scores

In the baseline analysis, higher weight-z-scores were associated with lower FA in both hemispheres (P<.001), foremost in the corpus callosum including forceps minor and major (P<.001), inferior-fronto-occipital fasciculi (P<.001) and superior longitudinal fasciculi (P<.02, eFigure 8). With higher weight-z-scores, MD was elevated in both brain hemispheres (P<.001), especially in the inferior-fronto-occipital fasciculi, corticospinal/pyramidal tracts and fornices (P<.001 for all). We also found higher RD in the right and left hemisphere (P<.001), most pronounced in the inferior-fronto-occipital fasciculi, corticospinal/pyramidal tracts, fornices and corpus callosum including forceps minor and major (P<.001 for all). With higher weight-z-scores, no axial diffusivity changes for the hemispheres were found, however higher AD was observed in the left corticospinal/pyramidal tract (P=.03), bilateral anterior thalamic radiations (P<.04) and inferior longitudinal fasciculi (P<.04). Furthermore, we also found lower AD in the right temporal superior longitudinal fasciculus (P=.03). Higher weight-z-scores were associated with pervasive reductions of ND in both hemispheres (P<.03), most pronounced in both inferior-fronto-occipital fasciculi, corpus callosum including forceps minor and major and right uncinate fasciculus (P<.001 for all). In the second-year follow-up cross-sectional analysis, results were similar except that, with higher weight-z-scores, there were also pervasive reductions of AD in both hemispheres (P<.001, eFigure 9).

Relationship of cortical morphology with weight-z-scores
At the baseline, higher weight-z-scores were associated with extensive reductions in cortical thickness in bilateral hemispheres (P<.001), especially frontal regions (eFigure 8), with similar patterns in the second-year follow-up cross-sectional analysis (eFigure 9).

Relationship of functional connectivity with weight-z-scores
With higher weight-z-scores at baseline, functional coupling was mostly reduced, including the correlations within the salience and cingulo-opercular networks, respectively, as well as the correlation of the salience with the cingulo-opercular network (P<.001 for all, eFigure 8). We also observed elevated functional connectivity strength between the default and dorsal attention network (P<.001), between the sensorimotor hand and visual network (P=.005), and between the dorsal and ventral attention network (P=.04). Similar results were observed in the second-year follow-up cross-sectional analysis (eFigure 9).

Comparison of MRI metrics between BMI categories

Comparison of WM microstructure and cytostructure between the overweight and normal weight group
Compared to the normal weight group ($5^{th} \leq$ BMI percentile < 85th)[32], the overweight group ($85^{th} \leq$ BMI percentile < 95th) showed lower FA in both hemispheres (P<.001), most pronounced in the corpus callosum including forceps minor (P<.001), inferior frontal superior frontal cortices (P<.003) and inferior-fronto-occipital fasciculi (P<.04, eFigure 10). Children with overweight also had higher RD in both brain hemispheres (P<.003), especially in the corticospinal/pyramidal tracts (P<.009), corpus callosum (P=.009) including forceps minor (P=.03) and major (P=.04), and inferior-fronto-occipital fasciculi (P<.02). Furthermore, in both hemispheres, lower ND was found (P≤.01). The ND reductions were observed in the right corticospinal/pyramidal tract (P=.01), right uncinate fasciculus (P=.01), right inferior-fronto-occipital fasciculus (P=.01) and corpus callosum including forceps minor (P=.01). The analysis at the second-year follow-up yielded similar results with extensive bilateral reductions of FA and AD in the overweight group compared to the normal weight group (P≤.002, eFigure 11). The eTable 2 summarizes ANOVA comparison of select neuroimaging metrics (with the strongest linear association) between BMI categories.

Comparison of cortical thickness between the overweight and normal weight group
In comparison to the normal weight group, the overweight group showed lower cortical thickness of both hemispheres (P=.009). The changes in cortical thickness were most pronounced in bilateral rostral middle frontal, superior frontal and lateral orbitofrontal regions (P<.001 for all, eFigure 12). At the second-year follow-up, no changes between the two groups regarding cortical thickness were observed. The eTable 2 summarizes ANOVA comparison of select neuroimaging metrics (with the strongest linear association) between BMI categories.

Comparison of functional connectivity between the overweight and normal weight group
No changes in functional coupling of networks were observed between the overweight and normal weight group at neither baseline nor second year. The eTable 2 summarizes ANOVA comparison of select neuroimaging metrics (with the strongest linear association) between BMI categories.

Comparison of WM microstructure and cytostructure between the obese and normal weight group
Similar to the overweight group, children with obesity ($95^{th} \leq$ BMI percentile)[32] showed extensive FA reductions in both hemispheres compared to the normal weight group, especially in both inferior-fronto-occipital fasciculi, right uncinate fasciculus and corpus callosum (P<.001 for all, eFigure 10). On the hemispheric level, no significant

© 2023 Kaltenhauser S et al. *JAMA Network Open.*

MD alterations between the obese and normal weight group were present, however, children with obesity had lower MD in several singular WM tracts including both cingulate gyri (P<.04), inferior frontal superior frontal cortices (P<.03), and corpus callosum including forceps minor (P<.0.4). For RD, again, no differences with respect to the hemispheres were found, but the obese group had higher RD in the right inferior-fronto-occipital fasciculus (P=.02) and right corticospinal/pyramidal tract (P=0.4). Extensive AD reductions were present in both hemispheres (P<.001), foremost in the corpus callosum including forceps minor and major (P<.001), both superior longitudinal fasciculi (P<.005) and cingulate gyri (P<.001). Obese children also had lower ND in both inferior-fronto-occipital fasciculi (P<.04). In the analysis at the second-year follow-up, we found – similar to the baseline analysis – extensive reductions in FA and AD of both hemispheres in the obese group compared to the normal weight group (P<.001 for all), but also lower averaged MD in both hemispheres (P<.05, eFigure 11). More specifically, we found lower MD in the corpus callosum including forceps minor (P<.04) and left cingulate gyrus (P=.005) among obese children. Furthermore, at second-year, lower ND was found in the right hemisphere (P=.05), namely in the right fornix (P=.03) and right inferior-fronto-occipital fasciculus (P=.04). The eTable 2 summarizes ANOVA comparison of select neuroimaging metrics (with the strongest linear association) between BMI categories.

Comparison of cortical thickness between the obese and normal weight group

Similar to the overweight group, the obese children had extensively reduced cortical thickness in both hemispheres (P<.001), especially in frontal cortical parcels, compared to the normal weight group at baseline (eFigure 12). At the second-year follow-up, these differences did not persist on the hemispheric level, but in singular cortical parcels: right rostral middle frontal (P<.001), right pars triangularis (P=.005) and right lateral orbitofrontal (P=.03, eFigure 12). The eTable 2 summarizes ANOVA comparison of select neuroimaging metrics (with the strongest linear association) between BMI categories.

Comparison of functional connectivity between the obese and normal weight group

Obese children had reduced functional connectivity at baseline compared to the normal weight group (eFigure 13). Most affected were the correlation within the salience and cingulo-opercular networks, respectively, as well as the correlation between the salience and cingulo-opercular network (P<.001 for all). The obese group showed higher connectivity only in the correlation between the sensorimotor-hand and visual network (P=.04). Similar to the baseline analysis, lower functional connectivity strength of the salience (P=.002) and cingulo-opercular (P=.04) within-network correlations were found in the second-year cross-sectional analysis (eFigure 13). The eTable 2 summarizes ANOVA comparison of select neuroimaging metrics (with the strongest linear association) between BMI categories.

Comparison of WM microstructure and cytostructure between the obese and overweight group

Comparing obese versus overweight children, we found extensive MD reductions in both hemispheres (P<.04, eFigure 10) that included both cingulate gyri (P<.02), corpus callosum and forceps minor (P<.007) and inferior frontal superior frontal cortices (P=.006). RD was reduced in singular WM tracts, namely, both inferior frontal superior frontal cortices (P=.03), left superior corticostriate-frontal cortex (P=.03) and forceps minor (P=.04). With respect to AD, we found extensive bilateral reductions in the obese compared to the overweight children (P<.03), most prominently, in both superior longitudinal fasciculi (P<.007), uncinate fasciculi (<.004), corpus callosum including forceps minor and major (P<.003) and cingulate gyri (P<.01). Repeating this analysis at second-year yielded lower AD only of the right hemisphere (P=.01, eFigure 11). The significant WM tracts were right uncinate fasciculus (P=.003), corpus callosum including forceps minor (P<.02), and right striatal inferior frontal cortex (P=.02). The eTable 2 summarizes ANOVA comparison of select neuroimaging metrics (with the strongest linear association) between BMI categories.

Comparison of cortical thickness between the obese and overweight group

In comparison to the overweight group, the obese group showed lower cortical thickness of singular cortical parcels, such as the medial (P=.004) and lateral orbitofrontal (P=.009, eFigure 12). Those parcels were mostly located in the frontal cortex. At the second-year, lower cortical thickness persisted only in the right rostral middle frontal parcel (P=.03, eFigure 12). The eTable 2 summarizes ANOVA comparison of select neuroimaging metrics (with the strongest linear association) between BMI categories.

Comparison of functional connectivity between the obese and overweight group

We found no differences in functional connectivity between the obese and overweight group at baseline or follow-up time points. The eTable 2 summarizes ANOVA comparison of select neuroimaging metrics (with the strongest linear association) between BMI categories.

© 2023 Kaltenhauser S et al. *JAMA Network Open.*

Exploration of linear versus polynomial association between MRI metrics and BMI

The BICs of the poylynomial models were lower than the BIC of the unadjusted linear model for AD and cortical thickness (eTable 3). With regard to FA, in three out of five analyzed WM tracts (corpus callosum, right inferior-fronto-occipital, right parietal superior longitudinal fasciculus), the BIC of the poynomial regression models were lower than the BIC of the univariate model. Using FA in the right uncinate fasciculus as the dependent variable, the two models had the same BIC (eTable 4 and eFigure 14). Moreover, for every MRI metric, the average MSE of the different polynomial models was not significantly different from the univariate model (eTable 5).

© 2023 Kaltenhauser S et al. *JAMA Network Open.*

eTable 1. Cohort Characteristics Across BMI Categories at the Second-Year Follow-up

	Total	Underweight[a]	Healthy weight[a]	Overweight[a]	Obese[a]	P-value
	n=1567	n=28 (1.8%)	n=1089 (69.5%)	n=244 (15.6%)	n=206 (13.2%)	
Age (months)	144.0 (7.7)	144.0 (8.8)	144.0 (7.6)	144.0 (7.9)	143.0 (8.2)	.690
Weight (kg)	47.6 (12.9)	32.3 (3.9)	41.9 (6.9)	55.4 (7.6)	70.5 (12.3)	<.001
Height (cm)	153.0 (8.4)	148.0 (8.0)	152.0 (8.1)	155.3 (8.5)	156.6 (8.2)	<.001
Waist circumference (cm)	72.0 (11.3)	61.9 (4.1)	67.2 (6.7)	78.7 (7.2)	91.2 (10.4)	<.001
BMI-z-score	0.4 (1.0)	-1.8 (0.1)	-0.1 (0.7)	1.3 (0.2)	2.0 (0.4)	<.001
BMI percentile	60.1 (29.5)	3.7 (1.0)	47.8 (23.4)	90.4 (3.1)	97.4 (1.5)	<.001
Puberty score[34, b]	2.0 (0.6)	1.6 (0.4)	1.9 (0.6)	2.2 (0.7)	2.3 (0.7)	<.001
Sex						<.001
Male	885 (56.5%)	18 (64.3%)	650 (59.7%)	125 (51.2%)	92 (44.7%)	
Female	682 (43.5%)	10 (35.7%)	439 (40.3%)	119 (48.8%)	114 (55.3%)	
Race/ethnicity						<.001
Asian	29 (1.9%)	2 (7.1%)	20 (1.8%)	4 (1.6%)	3 (1.5%)	
Black	131 (8.4%)	0 (0.0%)	73 (6.7%)	22 (9.0%)	36 (17.5%)	
Hispanic	306 (19.5%)	1 (3.6%)	167 (15.3%)	68 (27.9%)	70 (34.0%)	
White	983 (62.7%)	22 (78.6%)	754 (69.2%)	132 (54.1%)	75 (36.4%)	
Mixed/Other[c]	116 (7.4%)	3 (10.7%)	75 (6.9%)	17 (7.0%)	21 (10.2%)	
No answer	2 (0.1%)	0 (0.0%)	0 (0.0%)	1 (0.4%)	1 (0.5%)	
Parental education						<.001
<High School	80 (5.1%)	1 (3.6%)	41 (3.8%)	16 (6.6%)	22 (10.7%)	
High School/GED[d]	82 (5.2%)	0 (0.0%)	47 (4.3%)	11 (4.5%)	24 (11.7%)	
Some college[e]	362 (23.1%)	5 (17.9%)	208 (19.1%)	71 (29.1%)	78 (37.9%)	
Bachelor's Degree	442 (28.2%)	8 (28.6%)	335 (30.8%)	62 (25.4%)	37 (18.0%)	
Postgraduate	598 (38.2%)	14 (50.0%)	456 (41.9%)	84 (34.4%)	44 (21.4%)	
No answer	3 (0.2%)	0 (0.0%)	2 (0.2%)	0 (0.0%)	1 (0.5%)	
Handedness						.034
Right	1298 (82.8%)	25 (89.3%)	913 (83.8%)	204 (83.6%)	156 (75.7%)	
Left	87 (5.5%)	1 (3.6%)	59 (5.4%)	16 (6.6%)	11 (5.3%)	
Mixed	182 (11.6%)	2 (7.1%)	117 (10.7%)	24 (9.8%)	39 (18.9%)	
Family income[f]						<.001
<$5,000	32 (2.0%)	0 (0.0%)	8 (0.7%)	12 (4.9%)	12 (5.8%)	
$5,000 - $11,999	38 (2.4%)	1 (3.6%)	15 (1.4%)	9 (3.7%)	13 (6.3%)	
$12,000 - $15,999	24 (1.5%)	0 (0.0%)	15 (1.4%)	2 (0.8%)	7 (3.4%)	
$16,000 - $24,999	47 (3.0%)	0 (0.0%)	23 (2.1%)	9 (3.7%)	15 (7.3%)	
$25,000 - $34,999	78 (5.0%)	0 (0.0%)	50 (4.6%)	13 (5.3%)	15 (7.3%)	

© 2023 Kaltenhauser S et al. *JAMA Network Open.*

	Total	Underweight[a]	Healthy weight[a]	Overweight[a]	Obese[a]	P-value
$35,000 - $49,999	113 (7.2%)	3 (10.7%)	73 (6.7%)	11 (4.5%)	26 (12.6%)	
$50,000 - $74,999	209 (13.3%)	6 (21.4%)	143(13.1%)	34 (13.9%)	26 (12.6%)	
$75,000 - $99,999	249 (15.9%)	3 (10.7%)	175 (16.1%)	44 (18.0%)	27 (13.1%)	
$100,000 - $199,999	509 (32.5%)	9 (32.1%)	381 (35.0%)	73 (29.9%)	46 (22.3%)	
>$200,000	180 (11.5%)	4 (14.3%)	144 (13.2%)	25 (10.2%)	7 (3.4%)	
No answer	88 (5.6%)	2 (7.1%)	62 (5.7%)	12 (4.9%)	12 (5.8%)	

All numbers are presented as mean (standard deviation) or frequency (percentage)
a) BMI percentile cutoffs for the BMI categories: < 5th underweight, ≥ 5th and < 85th normal weight, ≥ 85th and < 95th overweight, ≥ 95th obese[32]
b) Overall pubertal development score: range from 1 (no development) to 4 (completed development)[34]
c) Mixed/Other: Alaska Native, American Indian, Guamanian, Native American, Native Hawaiian, Other Pacific Islander, Samoan, Other Race.
d) GED= General Educational Development
e) Some college / associate degree
f) Total combined family income for the past 12 months. This includes income (before taxes and deductions) from all sources. If separated/divorced, average of the two household incomes is used.

© 2023 Kaltenhauser S et al. *JAMA Network Open.*

eTable 2. Comparison of Neuroimaging Metrics Between BMI Categories

Baseline	Underweight[a]	Healthy weight[a]	Overweight[a]	Obese[a]	P-value
**n=4576**	**n=191 (4.2%)**	**n=3046 (66.6%)**	**n=683 (14.9%)**	**n=656 (14.3%)**	
Fractional anisotropy of the corpus callosum	0.59 (0.03)	0.59 (0.02)	0.58 (0.02)	0.58 (0.03)	<.001
Axial diffusivity of the right temporal superior longitudinal fasciculus	0.75 (0.02)	0.76 (0.02)	0.75 (0.02)	0.75 (0.02)	<.001
Neurite density of the right inferior-fronto-occipital fasciculus	0.63 (0.03)	0.63 (0.03)	0.63 (0.03)	0.62 (0.03)	<.001
Cortical thickness of the right rostralmiddlefrontal	2.70 (0.13)	2.69 (0.14)	2.66 (0.15)	2.64 (0.15)	<.001
Gordon network correlation within the salience network	0.43 (0.18)	0.41 (0.17)	0.39 (0.18)	0.37 (0.18)	<.001
**Second-year**	**Underweight[a]**	**Healthy weight[a]**	**Overweight[a]**	**Obese[a]**	**P-value**
**n=1567**	**n=28 (1.8%)**	**n=1089 (69.5%)**	**n=244 (15.6%)**	**n=206 (13.2%)**	
Fractional anisotropy of the corpus callosum	0.59 (0.02)	0.59 (0.03)	0.59 (0.03)	0.58 (0.03)	<.001
Axial diffusivity of the right temporal superior longitudinal fasciculus	0.75 (0.02)	0.75 (0.02)	0.75 (0.02)	0.74 (0.02)	<.001
Neurite density of the right inferior-fronto-occipital fasciculus	0.64 (0.03)	0.64 (0.03)	0.64 (0.03)	0.63 (0.03)	.001
Cortical thickness of the right rostralmiddlefrontal	2.60 (0.10)	2.60 (0.10)	2.59 (0.10)	2.55 (0.11)	<.001
Gordon network correlation within the salience network	0.47 (0.14)	0.39 (0.12)	0.39 (0.11)	0.36 (0.12)	<.001
**Interval changes from baseline to second-year follow-up[b]**	**Underweight[a]**	**Healthy weight[a]**	**Overweight[a]**	**Obese[a]**	**P-value**
**n=1567**	**n=51 (3.3%)**	**n=1114 (71.1%)**	**n=217 (13.9%)**	**n=185 (11.8%)**	
Fractional anisotropy of the corpus callosum	+0.01 (0.02)	+0.01 (0.02)	+0.00 (0.02)	+0.00 (0.02)	.003
Axial diffusivity of the right temporal superior longitudinal fasciculus	-0.01 (0.02)	-0.01 (0.02)	-0.01 (0.02)	-0.01 (0.02)	.027
Neurite density of the right inferior-fronto-occipital fasciculus	+0.01 (0.02)	+0.01 (0.02)	+0.01 (0.02)	+0.01 (0.02)	.032
Cortical thickness of the right rostralmiddlefrontal	-0.10 (0.08)	-0.09 (0.11)	-0.08 (0.10)	-0.08 (0.11)	.460
Gordon network correlation within the salience network	-0.05 (0.19)	-0.02 (0.17)	-0.01 (0.17)	0.00 (0.19)	.160

All numbers are presented as mean (standard deviation) or frequency (percentage)
a) BMI percentile cutoffs for the BMI categories: < 5th underweight, ≥ 5th and < 85th normal weight, ≥ 85th and < 95th overweight, ≥ 95th obese[32]
b) Interval changes from baseline to second-year follow-up: "+" indicates interval increase from baseline to second-year, "-" indicates interval decrease from baseline to second-year.

© 2023 Kaltenhauser S et al. *JAMA Network Open.*

eTable 3. Comparison of the Bayesian Information Criterion Between the Linear and Polynomial Model for the Most Significant Imaging Metrics

MRI metric	White matter tract/cortical region/Gordon network correlation	BIC of the univariate linear model	Best polynomial model	BIC of the best polynomial model
Fractional anisotropy	Corpus callosum	-24033.8	Y~ x + I(x^2) + I(x^3) + I(x^4)	-24021.3
Mean diffusivity	Right inferior-fronto-occipital fasciculus	-25466.0	Y ~ x + I(x^3) + I(x^4)	-25456.3
Radial diffusivity	Right corticospinal/pyramidal tract	-27117.9	Y ~ x + I(x^2) + I(x^4)	-27104.8
Axial diffusivity	Right temporal superior longitudinal fasciculus	-24389.8	Y ~ I(x^2) + I(x^3)	-24403.1
Neurite density	Right inferior-fronto-occipital fasciculus	-21914.2	Y ~ I(x^2) + I(x^3) + I(x^4) + I(x^5)	-21896.6
Cortical thickness	Right rostralmiddlefrontal	-5479.4	Y ~ x + I(x^2) + I(x^5)	-5481.5
Gordon network correlation	Correlation within the salience network	-3459.3	Y ~ x + I(x^2)	-3454.3

Comparison of the Bayesian information criterion (BIC) between the linear model and best polynomial model selected via backwards selection for the most significant white matter tract/cortical region/functional correlation of each MRI metric. We selected the best polynomial model by fitting a polynomial relationship between MRI metrics and BMI-z-scores as the explanatory variable including up to the 5th degree of polynomial and using backwards variable selection on the degrees. Y MRI-metric, x BMI-z-score.

© 2023 Kaltenhauser S et al. *JAMA Network Open.*

eTable 4. Comparison of the Bayesian Information Criterion Between the Linear and Polynomial Models for the Most Significant WM Tract Fractional Anisotropy

**Fractional anisotropy**	**BIC of the univariate linear model**	**Best polynomial model**	**BIC of the best polynomial model**
Corpus callosum	-24034.9	y ~ I(x^3)	-24045.8
Right inferior-fronto-occipital fasciculus	-23952.7	y ~ I(x^5)	-23971.5
Right temporal superior longitudinal fasciculus	-23379.5	y ~ I(x^3) + I(x^4) + I(x^5)	-23371.5
Right parietal superior longitudinal fasciculus	-23500.4	y ~ I(x^2)	-23504.4
Right uncinate fasciculus	-23273.2	y ~ x + I(x^2)	-23273.2

Comparison of the Bayesian information criterion (BIC) between the univariate linear model and the best polynomial model selected via backwards selection for the five most significant white matter tracts of fractional anisotropy in the baseline cross-sectional analysis. We selected the best polynomial model by fitting a polynomial relationship between fractional anistropy metrics and BMI percentiles as the explanatory variable including up to the 5th degree of polynomial and using backwards variable selection on the degrees. Y fractional anistropy, x BMI percentile.

© 2023 Kaltenhauser S et al. *JAMA Network Open.*

eTable 5. Comparison of the Mean Squared Error (MSE) of the Linear and Different Polynomial Degree Models

Average MSE of 35 white matter tracts for	**y ~ x**	**y ~ I(x^2)**	**y ~ I(x^3)**	**y ~ I(x^4)**	**y ~ I(x^5)**	**P-value**
Fractional anisotropy	0.0008	0.0008	0.0008	0.0008	0.0008	>.99
Mean diffusivity	0.0004	0.0004	0.0004	0.0004	0.0004	>.99
Radial diffusivity	0.0005	0.0005	0.0005	0.0005	0.0005	>.99
Axial diffusivity	0.0011	0.0011	0.0011	0.0011	0.0011	>.99
Neurite density	0.0009	0.0009	0.0009	0.0009	0.0009	>.99
Average MSE of 68 regions for cortical thickness	0.0217	0.0217	0.0217	0.0217	0.0217	>.99
Average MSE of 91 Gordon network correlations	0.0044	0.0044	0.0044	0.0045	0.0045	>.99

© 2023 Kaltenhauser S et al. *JAMA Network Open.*

eFigure 1. Image Inclusion and Exclusion Process

(A) Image inclusion/exclusion process at baseline

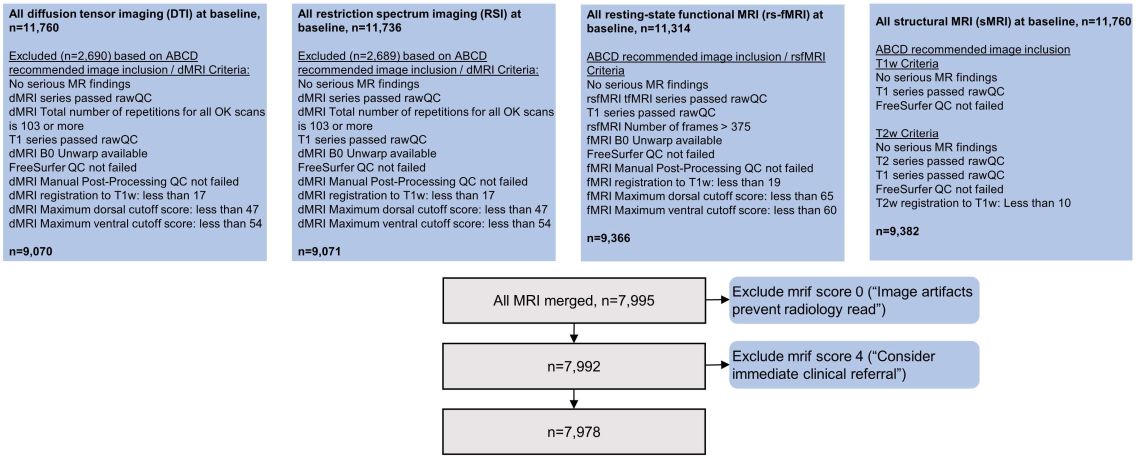

(B) Image inclusion/exclusion process at second-year

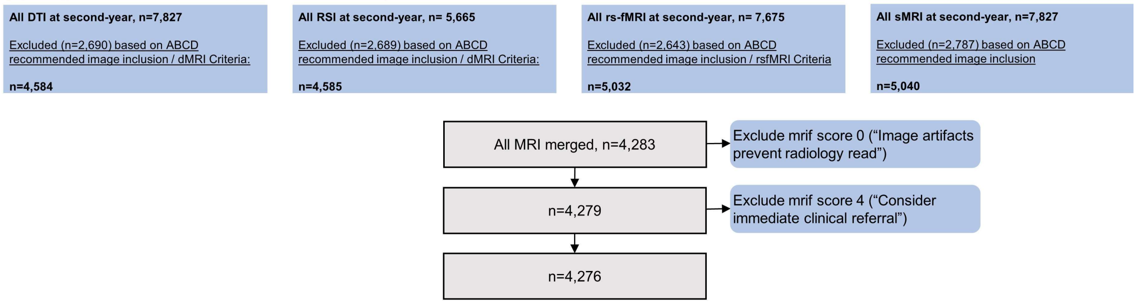

Image inclusion/exclusion at (A) baseline and (B) second-year was based on ABCD Recommended image inclusion protocol as well as ABCD MRI findings.

© 2023 Kaltenhauser S et al. *JAMA Network Open.*

eFigure 2. Association of WM Microstructural and Cytostructural Markers With Higher BMI *z* Scores and Waist Circumference in the Baseline Cross-sectional Analysis

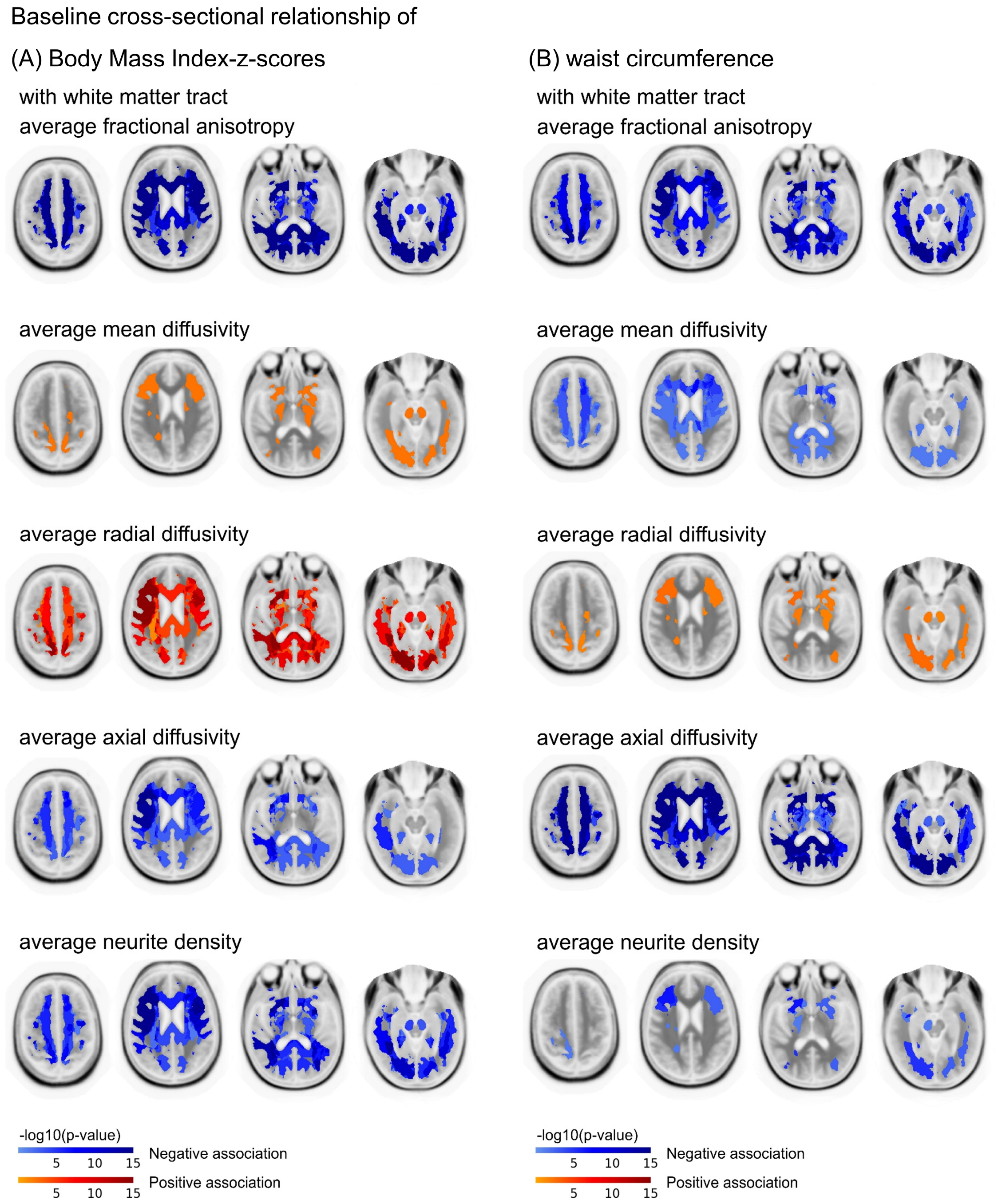

The white matter tracts where fractional anisotropy, mean, radial and axial diffusivity, and neurite density were significantly associated with children's higher (A) BMI-z-scores and (B) waist-circumference (after correction for age, sex, race/ethnicity, handedness, socioeconomic status, MRI scanner, puberty and intracranial volume) are color-coded as blue representing negative association and red representing positive association.

© 2023 Kaltenhauser S et al. *JAMA Network Open.*

eFigure 3. Association of WM Microstructural and Cytostructural Markers With Higher BMI *z* Scores and Waist Circumference in the Second-Year Follow-up Cross-sectional Analysis

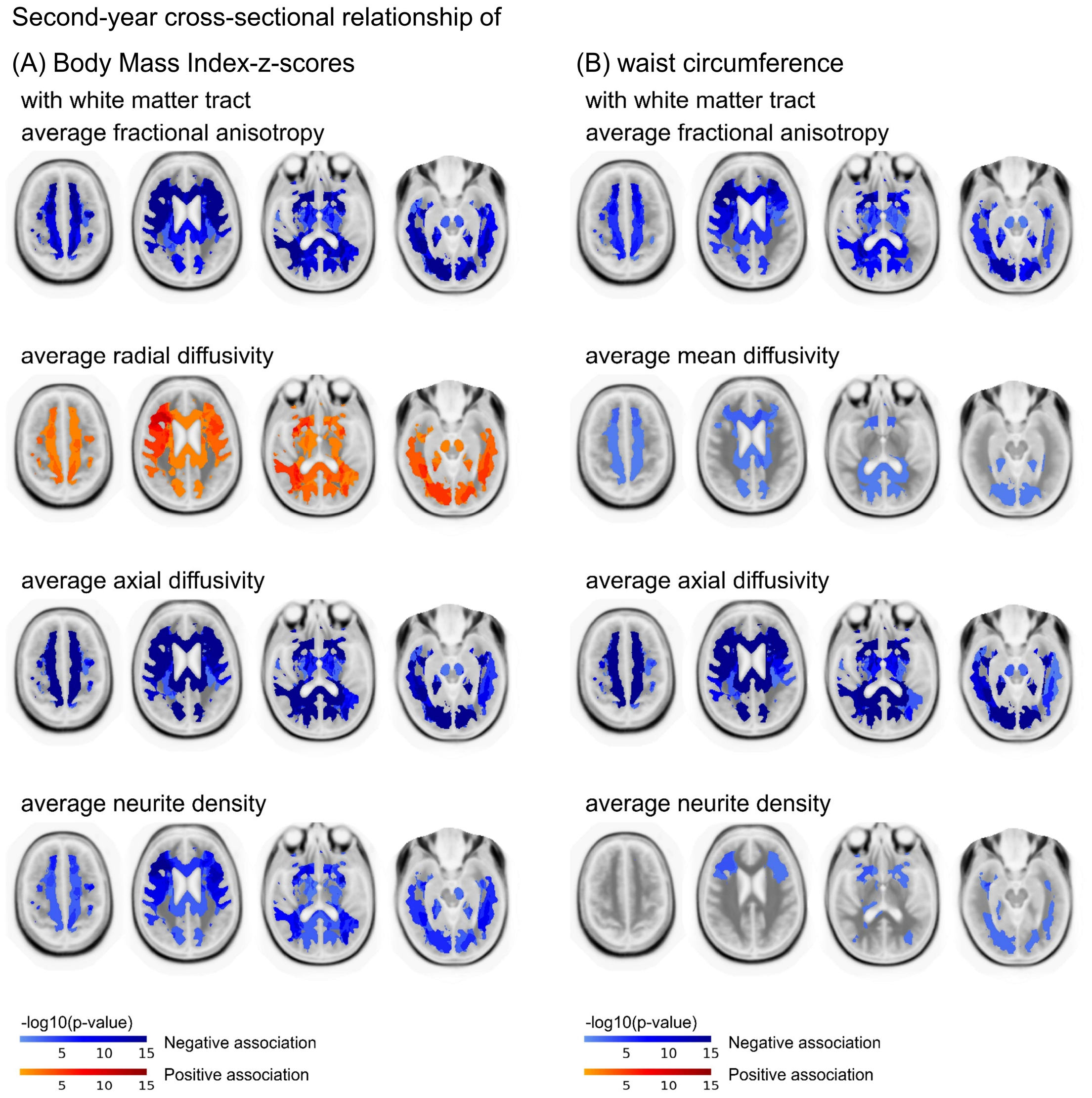

The white matter tracts where fractional anisotropy, mean, radial and axial diffusivity, and neurite density were significantly associated with children's higher (A) BMI-z-scores and (B) waist-circumference (after correction for age, sex, race/ethnicity, handedness, socioeconomic status, MRI scanner, puberty and intracranial volume) are color-coded as blue representing negative association and red representing positive association.

eFigure 4. Association of Brain Cortex Morphology With Higher BMI *z* Scores and Waist Circumference in the Second-Year Follow-up Cross-sectional Analysis

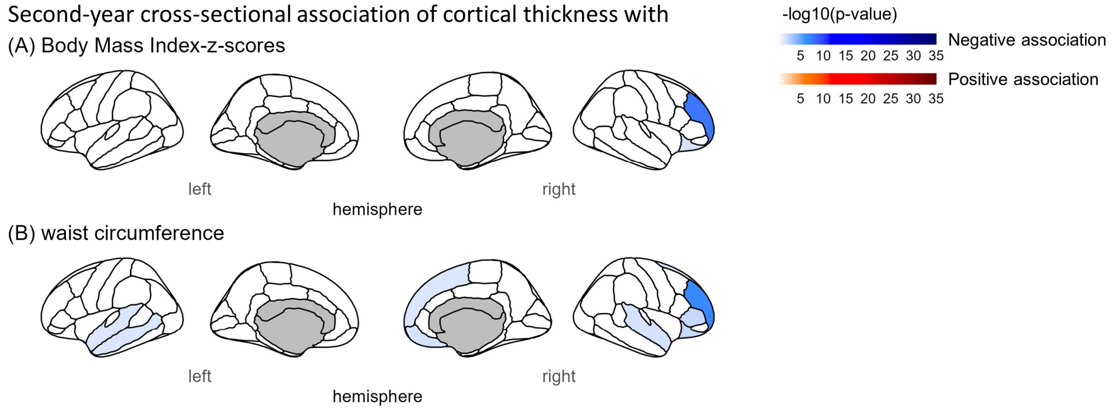

Several regions had a significant negative association of cortical thickness with children's (A) BMI-z-scores and (B) waist-circumference – blue coloring – after correction for age, sex, race/ethnicity, handedness, socioeconomic status, MRI scanner, puberty and intracranial volume.

© 2023 Kaltenhauser S et al. *JAMA Network Open.*

eFigure 5. Association of Functional Connectivity With BMI *z* Scores and Waist Circumference in the Baseline Cross-sectional Analysis

Baseline cross-sectional association of functional network correlations with

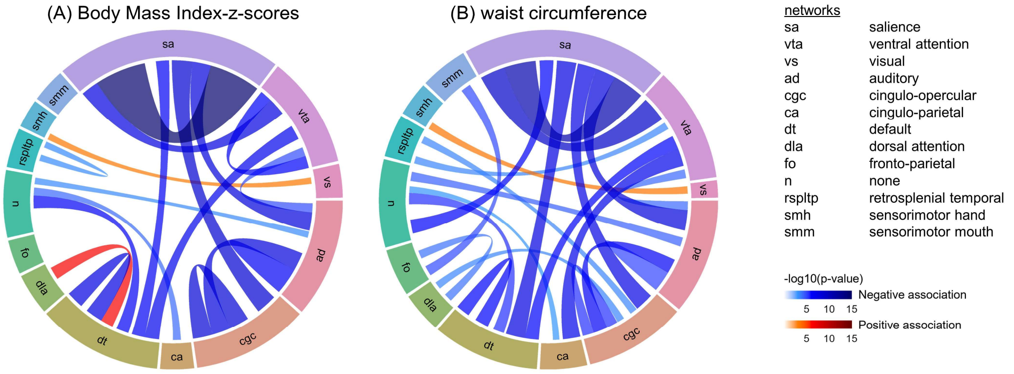

The connectogram depicts the association of higher (A) BMI-z-scores and (B) waist-circumference with averaged intra- and inter-network correlations from 13 pre-defined resting-state functional MRI networks[1,30]. Blue coloring represents negative association and red coloring positive association.

eFigure 6. Association of Functional Connectivity With BMI *z* Scores and Waist Circumference in the Second-Year Follow-up Cross-sectional Analysis

Second-year cross-sectional association of functional network correlations with

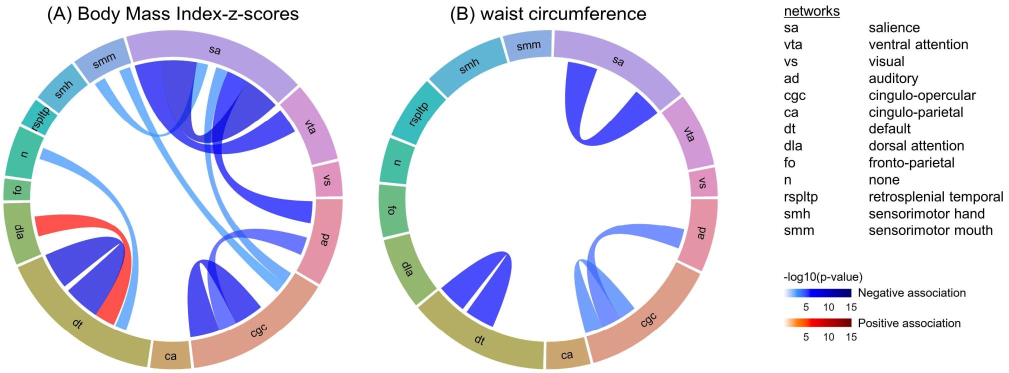

The connectogram depicts the significant association of higher (A) BMI-z-scores and (B) waist-circumference with averaged intra- and inter-network correlations from 13 pre-defined resting-state functional MRI networks[1,30] – after correction for age, sex, race/ethnicity, handedness, socioeconomic status, MRI scanner, puberty and intracranial volume. Blue coloring represents negative association and red represents positive association.

© 2023 Kaltenhauser S et al. *JAMA Network Open.*

eFigure 7. Interval Changes of Cortical Thickness, WM Microstructure and Cytostructure, and Functional Connectivity From Baseline to Second-Year Follow-up

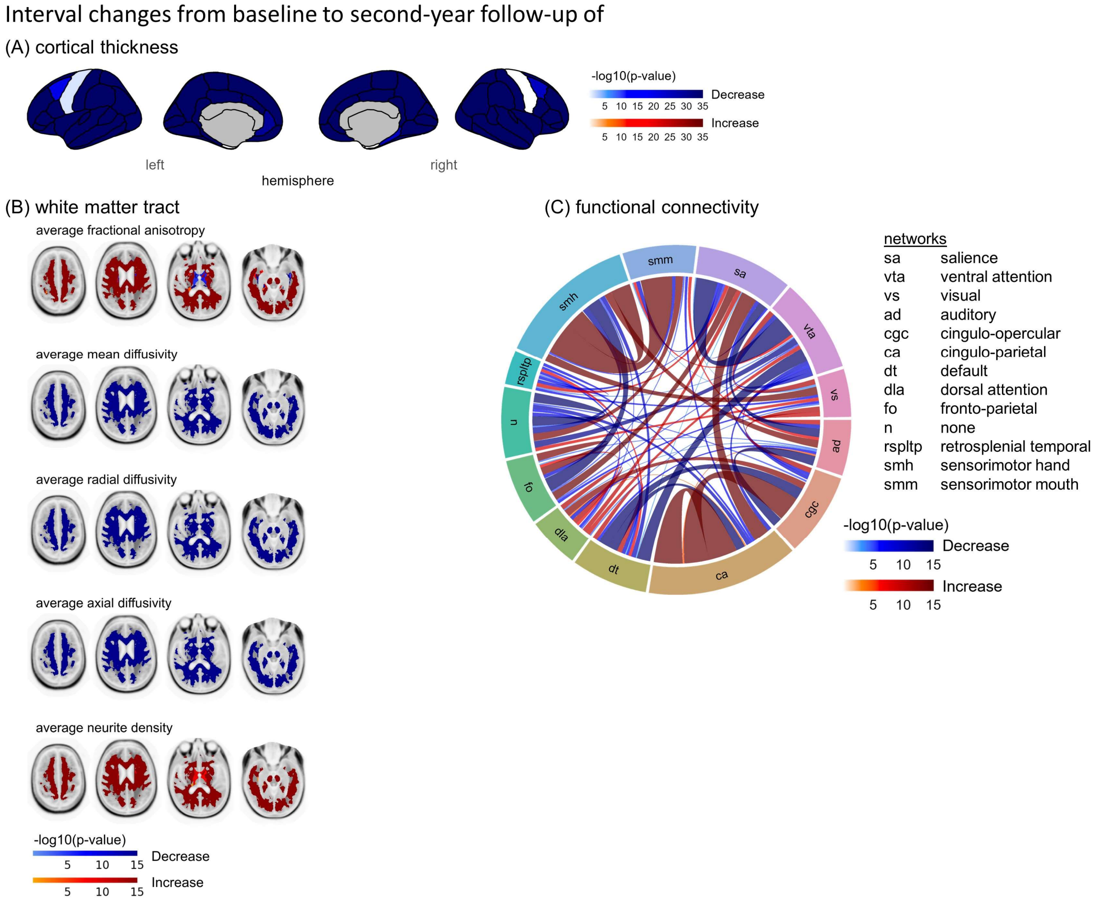

Only (A) cortical regions, (B) white matter tracts, and (C) functional connectivity correlations with significant mean differences from baseline to second-year are color-coded, with blue representing interval decrease and red representing interval increase.

eFigure 8. Association of Cortical Thickness, WM Tract Microstructural and Cytostructural Markers, and Functional Connectivity With Weight *z* Scores in the Baseline Cross-sectional Analysis

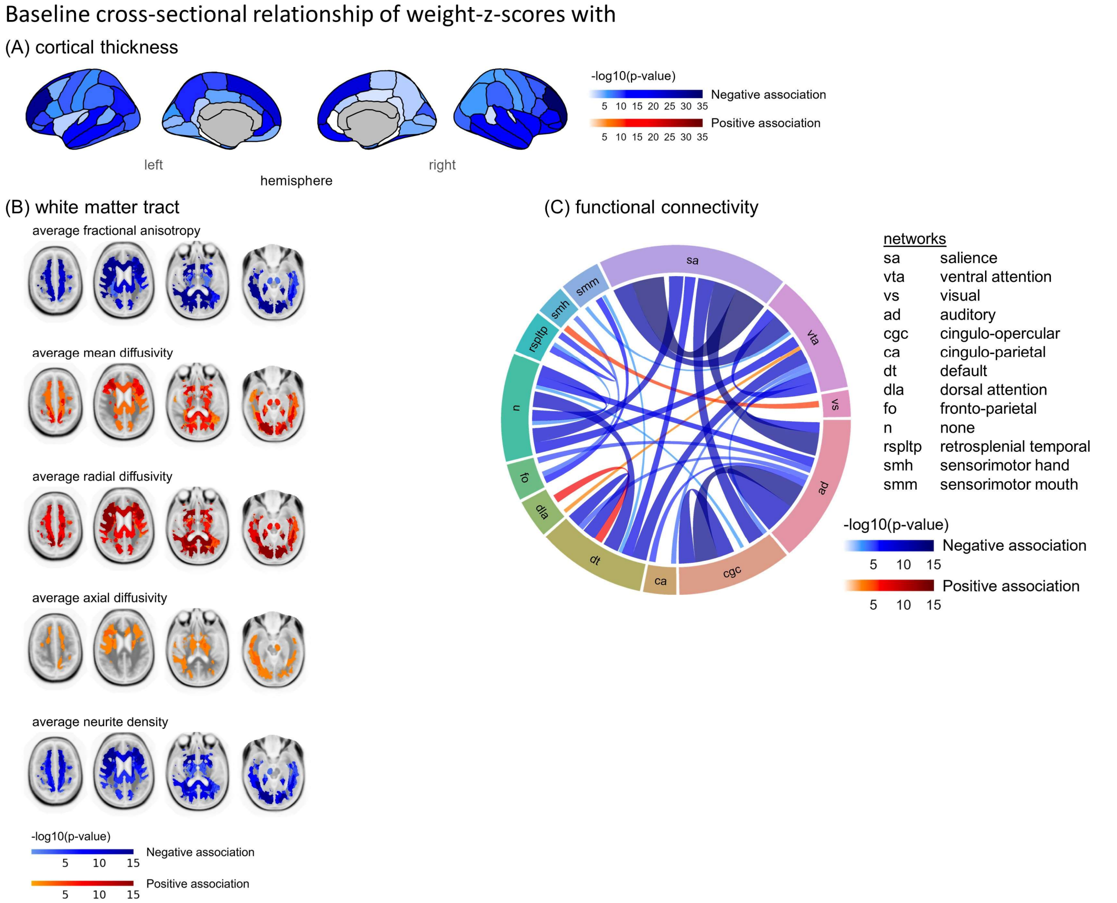

Relationship of (A) cortex morphology, (B) WM tract microstructural and cytostructural markers, and (C) functional connectivity with higher weight-z-scores at baseline – after correction for age, sex, race/ethnicity, handedness, socioeconomic status, MRI scanner, puberty and intracranial volume. Only regions with significant associations are color-coded. Blue coloring represents negative association, red coloring positive association.

© 2023 Kaltenhauser S et al. *JAMA Network Open.*

eFigure 9. Association of Cortical Thickness, WM Tract Microstructural and Cytostructural Markers, and Functional Connectivity With Weight *z* Scores in the Second-Year Follow-up Cross-sectional Analysis

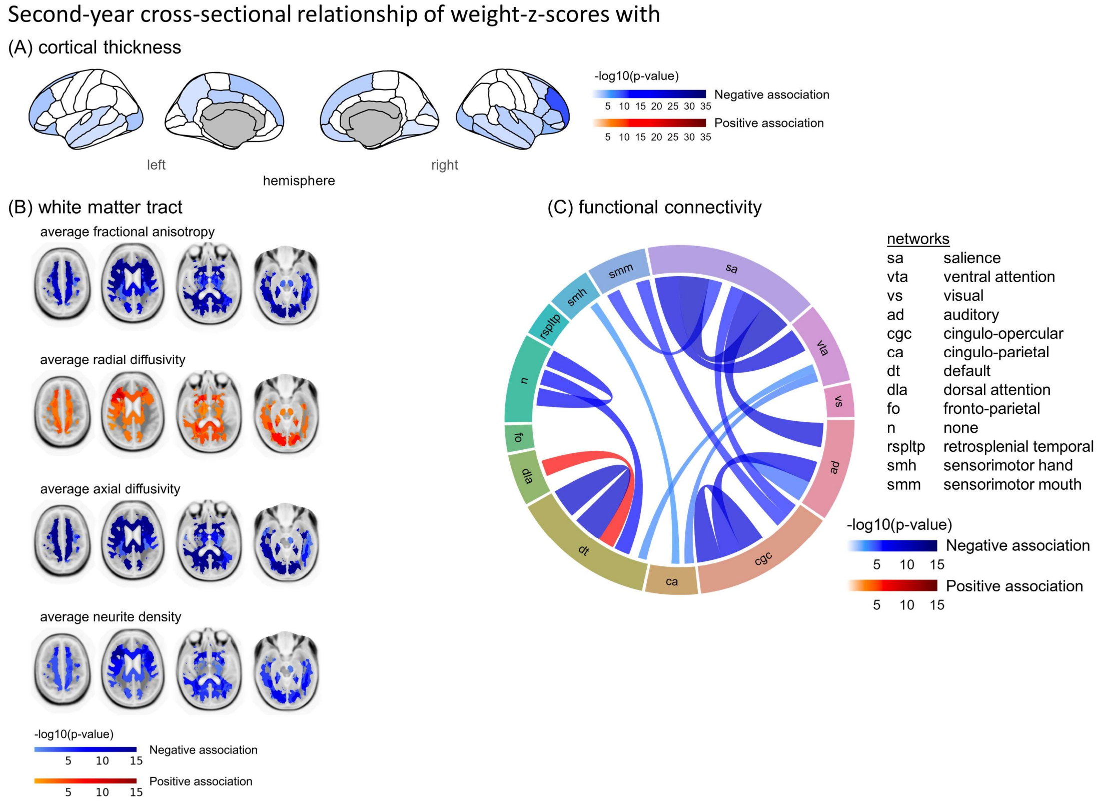

Relationship of (A) cortex morphology, (B) WM tract microstructural and cytostructural markers, and (C) functional connectivity with higher weight-z-scores at the second-year follow-up – after correction for age, sex, race/ethnicity, handedness, socioeconomic status, MRI scanner, puberty and intracranial volume. Only regions with significant associations are color-coded. Blue coloring represents negative association, red coloring positive association.

© 2023 Kaltenhauser S et al. *JAMA Network Open.*

eFigure 10. Significant Differences in WM Microstructure and Cytostructure Between BMI Categories in the Baseline Cross-sectional Analysis

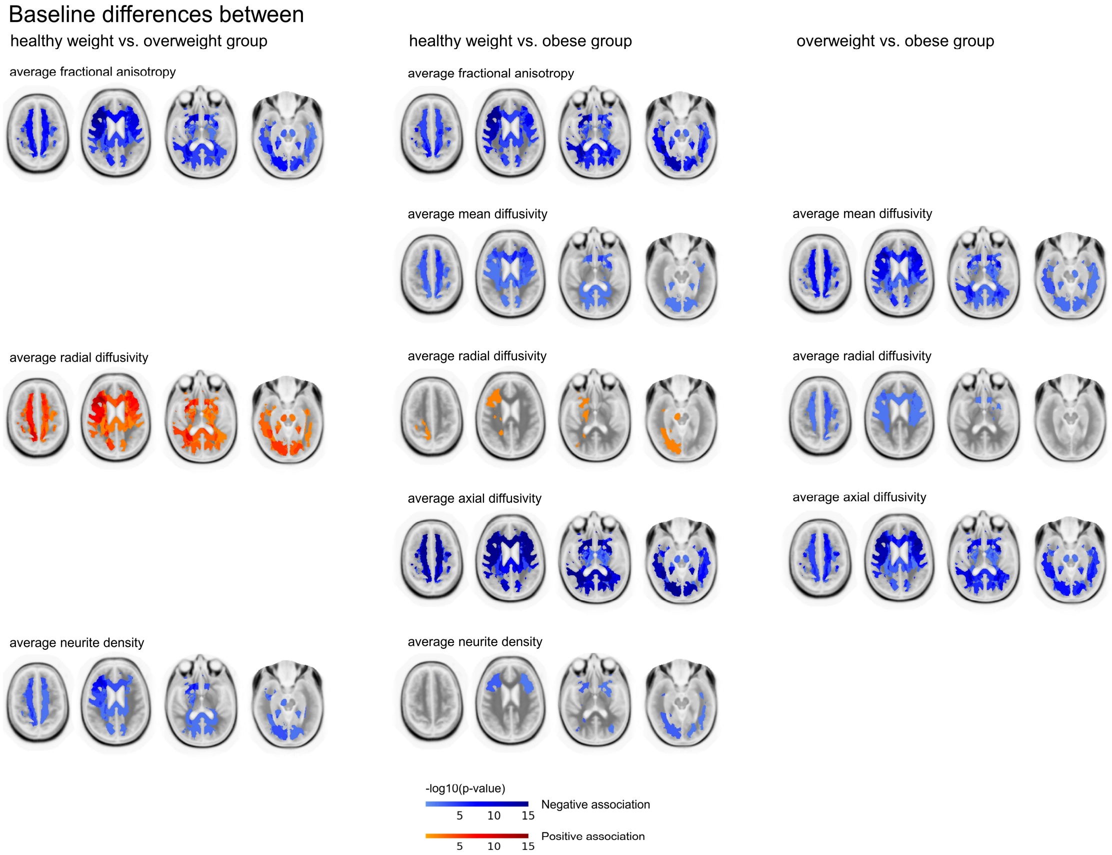

Two BMI groups were compared to each other using the group with the lower BMI as reference, respectively. Blue coloring represents negative association, red coloring positive association. Analyses were corrected for age, sex, race/ethnicity, handedness, socioeconomic status, MRI scanner, puberty and intracranial volume.

© 2023 Kaltenhauser S et al. *JAMA Network Open.*

eFigure 11. Significant Differences in WM Microstructure and Cytostructure Between BMI Categories in the Second-Year Follow-up Cross-sectional Analysis

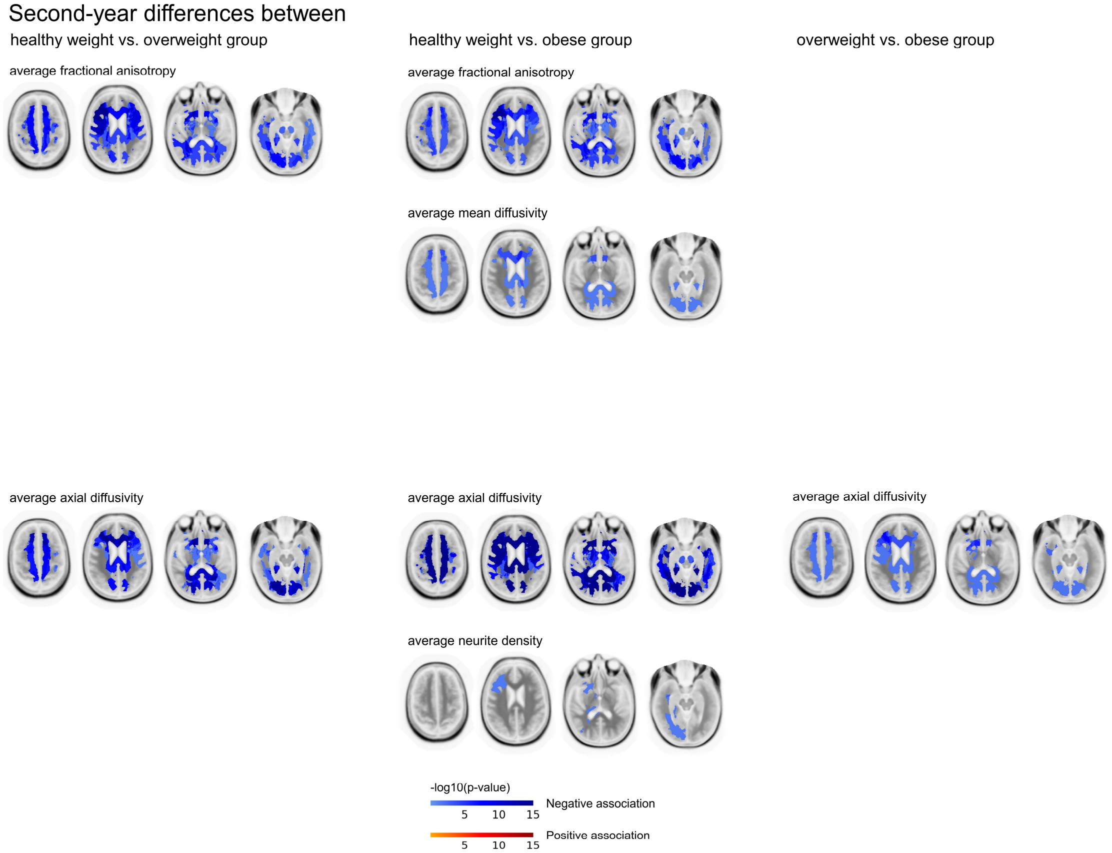

Two BMI groups were compared to each other using the group with the lower BMI as reference, respectively. Blue coloring represents negative association, red coloring positive association. Analyses were corrected for age, sex, race/ethnicity, handedness, socioeconomic status, MRI scanner, puberty and intracranial volume.

© 2023 Kaltenhauser S et al. *JAMA Network Open.*

eFigure 12. Significant Differences in Brain Cortex Morphology Between BMI Categories in the Baseline and Second-Year Follow-up Cross-sectional Analyses

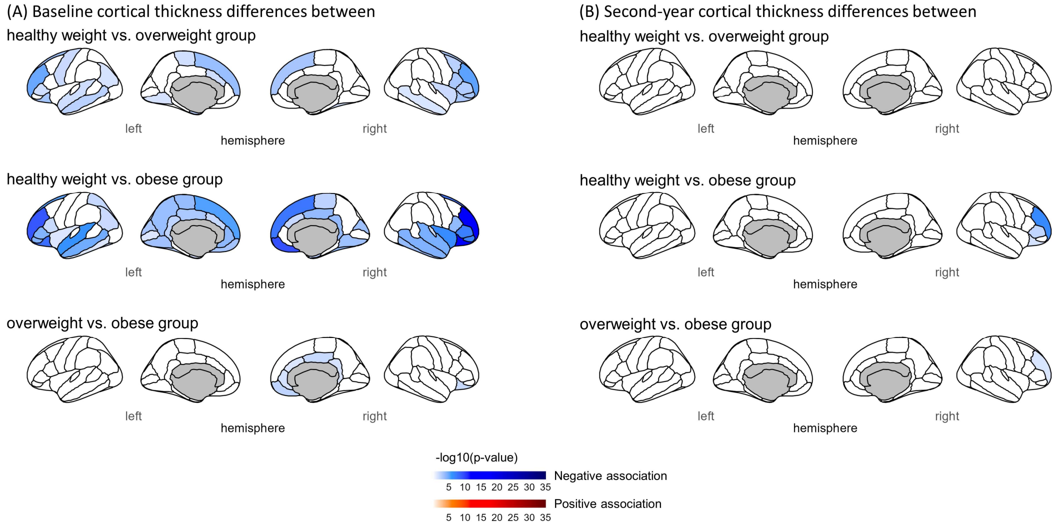

Two BMI groups were compared to each other using the group with the lower BMI as reference, respectively. Blue coloring represents negative association, red coloring positive association. Analyses were corrected for age, sex, race/ethnicity, handedness, socioeconomic status, MRI scanner, puberty and intracranial volume.

© 2023 Kaltenhauser S et al. *JAMA Network Open.*

eFigure 13. Significant Differences in Functional Connectivity Between the Groups With Normal Weight and Obesity in the Baseline and Second-Year Cross-sectional Analyses

Healthy weight vs. obese group differences of functional network correlations at

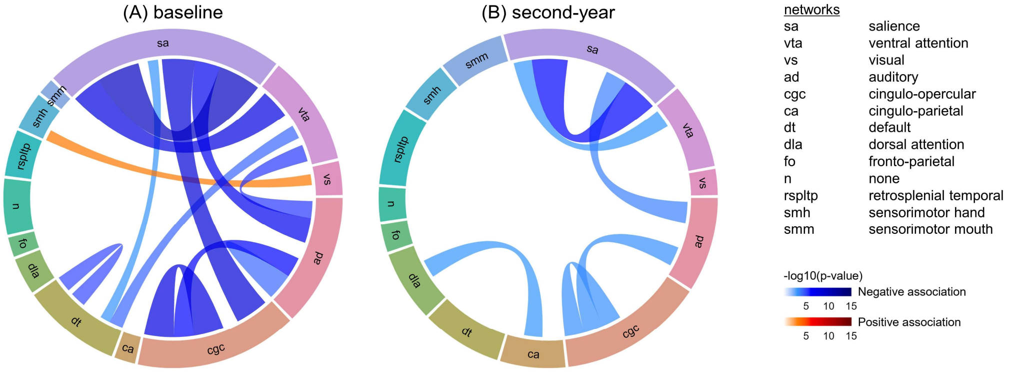

Two BMI groups were compared to each other using the group with the lower BMI as reference, respectively. Blue coloring represents negative association, red coloring positive association. Analyses were corrected for age, sex, race/ethnicity, handedness, socioeconomic status, MRI scanner, puberty and intracranial volume.

eFigure 14. Association of Average Fractional Anisotropy With BMI *z* Scores in the Baseline Cross-sectional Analysis

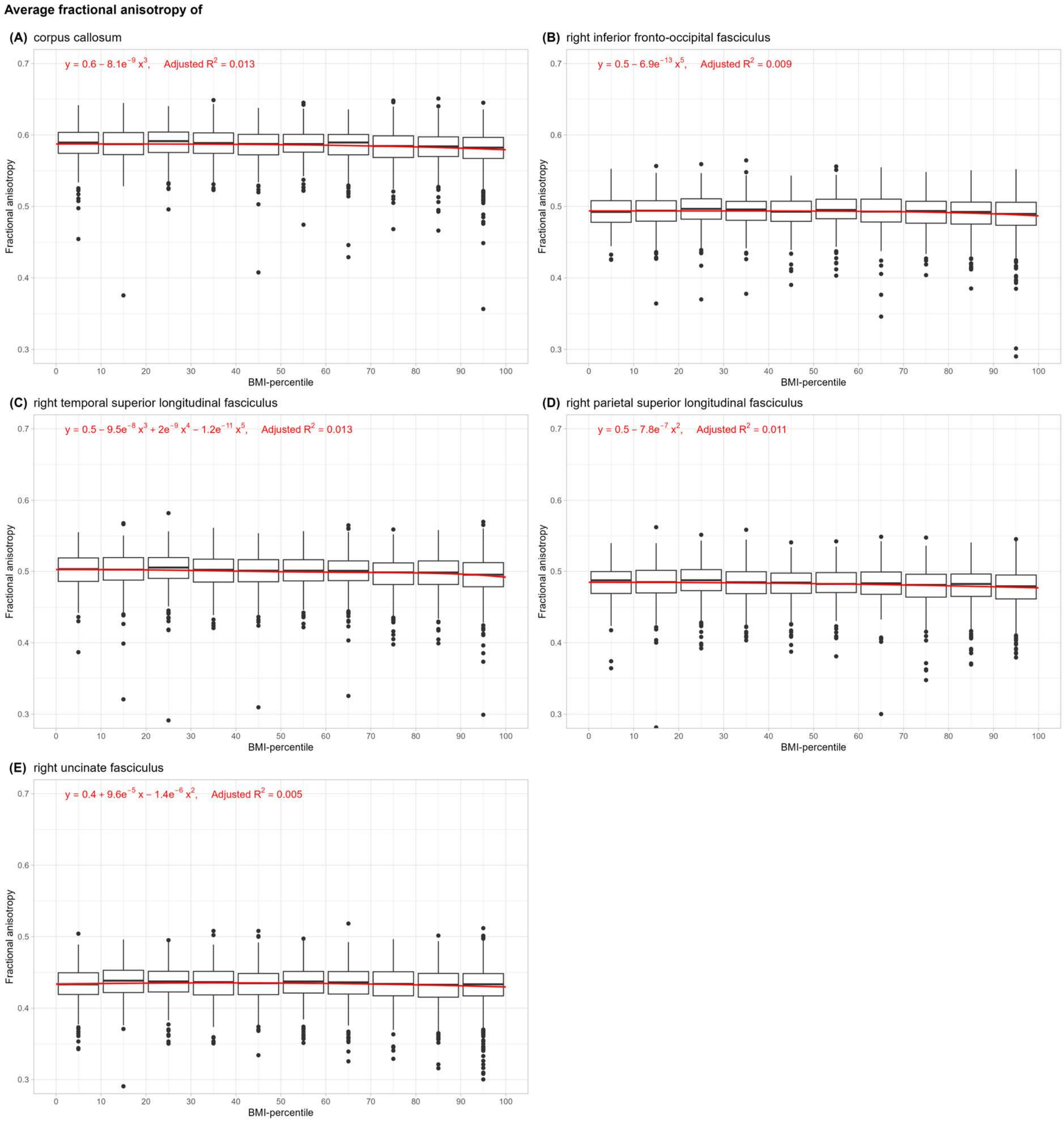

The associations of average fractional anisotropy of the five most significant tracts in the baseline cross-sectional analysis with BMI percentiles are shown. We selected the best polynomial model by fitting a polynomial relationship between MRI metrics and BMI percentiles as the explanatory variable including up to the 5th degree of polynomial and using backwards variable selection on the degrees.

© 2023 Kaltenhauser S et al. *JAMA Network Open.*

eReferences

1. Hagler DJ, Jr., Hatton S, Cornejo MD, et al. Image processing and analysis methods for the Adolescent Brain Cognitive Development Study. *Neuroimage*. Nov 15 2019;202:116091. doi:10.1016/j.neuroimage.2019.116091
2. Jovicich J, Czanner S, Greve D, et al. Reliability in multi-site structural MRI studies: effects of gradient non-linearity correction on phantom and human data. *Neuroimage*. Apr 1 2006;30(2):436-43. doi:10.1016/j.neuroimage.2005.09.046
3. Wells WM, 3rd, Viola P, Atsumi H, Nakajima S, Kikinis R. Multi-modal volume registration by maximization of mutual information. *Med Image Anal*. Mar 1996;1(1):35-51. doi:10.1016/s1361-8415(01)80004-9
4. Segonne F, Dale AM, Busa E, et al. A hybrid approach to the skull stripping problem in MRI. *Neuroimage*. Jul 2004;22(3):1060-75. doi:10.1016/j.neuroimage.2004.03.032
5. Dale AM, Fischl B, Sereno MI. Cortical surface-based analysis. I. Segmentation and surface reconstruction. *Neuroimage*. Feb 1999;9(2):179-94. doi:10.1006/nimg.1998.0395
6. Fischl B, Liu A, Dale AM. Automated manifold surgery: constructing geometrically accurate and topologically correct models of the human cerebral cortex. *IEEE Trans Med Imaging*. Jan 2001;20(1):70-80. doi:10.1109/42.906426
7. Segonne F, Pacheco J, Fischl B. Geometrically accurate topology-correction of cortical surfaces using nonseparating loops. *IEEE Trans Med Imaging*. Apr 2007;26(4):518-29. doi:10.1109/TMI.2006.887364
8. Dale AM, Sereno MI. Improved Localizadon of Cortical Activity by Combining EEG and MEG with MRI Cortical Surface Reconstruction: A Linear Approach. *J Cogn Neurosci*. Spring 1993;5(2):162-76. doi:10.1162/jocn.1993.5.2.162
9. Fischl B, Dale AM. Measuring the thickness of the human cerebral cortex from magnetic resonance images. *Proc Natl Acad Sci U S A*. Sep 26 2000;97(20):11050-5. doi:10.1073/pnas.200033797
10. Fischl B, Sereno MI, Tootell RB, Dale AM. High-resolution intersubject averaging and a coordinate system for the cortical surface. *Hum Brain Mapp*. 1999;8(4):272-84. doi:10.1002/(sici)1097-0193(1999)8:4<272::aid-hbm10>3.0.co;2-4
11. Fischl B, Salat DH, Busa E, et al. Whole brain segmentation: automated labeling of neuroanatomical structures in the human brain. *Neuron*. Jan 31 2002;33(3):341-55. doi:10.1016/s0896-6273(02)00569-x
12. Desikan RS, Segonne F, Fischl B, et al. An automated labeling system for subdividing the human cerebral cortex on MRI scans into gyral based regions of interest. *Neuroimage*. Jul 1 2006;31(3):968-80. doi:10.1016/j.neuroimage.2006.01.021
13. Zhuang J, Hrabe J, Kangarlu A, et al. Correction of eddy-current distortions in diffusion tensor images using the known directions and strengths of diffusion gradients. *J Magn Reson Imaging*. Nov 2006;24(5):1188-93. doi:10.1002/jmri.20727
14. Hagler DJ, Jr., Ahmadi ME, Kuperman J, et al. Automated white-matter tractography using a probabilistic diffusion tensor atlas: Application to temporal lobe epilepsy. *Hum Brain Mapp*. May 2009;30(5):1535-47. doi:10.1002/hbm.20619
15. Leemans A, Jones DK. The B-matrix must be rotated when correcting for subject motion in DTI data. *Magn Reson Med*. Jun 2009;61(6):1336-49. doi:10.1002/mrm.21890
16. Chang LC, Jones DK, Pierpaoli C. RESTORE: robust estimation of tensors by outlier rejection. *Magn Reson Med*. May 2005;53(5):1088-95. doi:10.1002/mrm.20426
17. Andersson JL, Skare S, Ashburner J. How to correct susceptibility distortions in spin-echo echo-planar images: application to diffusion tensor imaging. *Neuroimage*. Oct 2003;20(2):870-88. doi:10.1016/S1053-8119(03)00336-7
18. Smith SM, Jenkinson M, Woolrich MW, et al. Advances in functional and structural MR image analysis and implementation as FSL. *Neuroimage*. 2004;23 Suppl 1:S208-19. doi:10.1016/j.neuroimage.2004.07.051
19. Basser PJ, Mattiello J, LeBihan D. MR diffusion tensor spectroscopy and imaging. *Biophys J*. Jan 1994;66(1):259-67. doi:10.1016/S0006-3495(94)80775-1
20. Pierpaoli C, Jezzard P, Basser PJ, Barnett A, Di Chiro G. Diffusion tensor MR imaging of the human brain. *Radiology*. Dec 1996;201(3):637-48. doi:10.1148/radiology.201.3.8939209
21. Le Bihan D, Mangin JF, Poupon C, et al. Diffusion tensor imaging: concepts and applications. *J Magn Reson Imaging*. Apr 2001;13(4):534-46. doi:10.1002/jmri.1076
22. White NS, Leergaard TB, D'Arceuil H, Bjaalie JG, Dale AM. Probing tissue microstructure with restriction spectrum imaging: Histological and theoretical validation. *Hum Brain Mapp*. Feb 2013;34(2):327-46. doi:10.1002/hbm.21454
23. White NS, McDonald C, Farid N, et al. Diffusion-weighted imaging in cancer: physical foundations and applications of restriction spectrum imaging. *Cancer Res*. Sep 1 2014;74(17):4638-52. doi:10.1158/0008-5472.CAN-13-3534

© 2023 Kaltenhauser S et al. *JAMA Network Open.*

24. White NS, McDonald CR, Farid N, Kuperman JM, Kesari S, Dale AM. Improved conspicuity and delineation of high-grade primary and metastatic brain tumors using "restriction spectrum imaging": quantitative comparison with high B-value DWI and ADC. *AJNR Am J Neuroradiol*. May 2013;34(5):958-64, S1. doi:10.3174/ajnr.A3327
25. Cox RW. AFNI: software for analysis and visualization of functional magnetic resonance neuroimages. *Comput Biomed Res*. Jun 1996;29(3):162-73. doi:10.1006/cbmr.1996.0014
26. Power JD, Mitra A, Laumann TO, Snyder AZ, Schlaggar BL, Petersen SE. Methods to detect, characterize, and remove motion artifact in resting state fMRI. *Neuroimage*. Jan 1 2014;84:320-41. doi:10.1016/j.neuroimage.2013.08.048
27. Satterthwaite TD, Wolf DH, Loughead J, et al. Impact of in-scanner head motion on multiple measures of functional connectivity: relevance for studies of neurodevelopment in youth. *Neuroimage*. Mar 2012;60(1):623-32. doi:10.1016/j.neuroimage.2011.12.063
28. Hallquist MN, Hwang K, Luna B. The nuisance of nuisance regression: spectral misspecification in a common approach to resting-state fMRI preprocessing reintroduces noise and obscures functional connectivity. *Neuroimage*. Nov 15 2013;82:208-25. doi:10.1016/j.neuroimage.2013.05.116
29. Power JD, Barnes KA, Snyder AZ, Schlaggar BL, Petersen SE. Spurious but systematic correlations in functional connectivity MRI networks arise from subject motion. *Neuroimage*. Feb 1 2012;59(3):2142-54. doi:10.1016/j.neuroimage.2011.10.018
30. Gordon EM, Laumann TO, Adeyemo B, Huckins JF, Kelley WM, Petersen SE. Generation and Evaluation of a Cortical Area Parcellation from Resting-State Correlations. *Cereb Cortex*. Jan 2016;26(1):288-303. doi:10.1093/cercor/bhu239
31. Van Dijk KR, Hedden T, Venkataraman A, Evans KC, Lazar SW, Buckner RL. Intrinsic functional connectivity as a tool for human connectomics: theory, properties, and optimization. *J Neurophysiol*. Jan 2010;103(1):297-321. doi:10.1152/jn.00783.2009
32. Kuczmarski RJ, Ogden CL, Guo SS, et al. 2000 CDC Growth Charts for the United States: methods and development. *Vital Health Stat 11*. May 2002;(246):1-190.
33. Schwarz G. Estimating the Dimension of a Model. *The Annals of Statistics*. 1978;6(2):461-464.
34. Petersen AC, Crockett L, Richards M, Boxer A. A self-report measure of pubertal status: Reliability, validity, and initial norms. *J Youth Adolesc*. Apr 1988;17(2):117-33. doi:10.1007/BF01537962

© 2023 Kaltenhauser S et al. *JAMA Network Open.*
