# Supplementary material for: Association of Body Mass Index and Waist Circumference With Imaging Metrics of Brain Integrity and Functional Connectivity in Children Aged 9 to 10 Years in the US, 2016-2018
Source: JAMA Netw Open. 2023 May 18;6(5):e2314193. doi: 10.1001/jamanetworkopen.2023.14193 (PMC10196880; doi:10.1001/jamanetworkopen.2023.14193)
Supplement: Supplement 2. — Data Sharing Statement [file jamanetwopen-e2314193-s002.pdf]

Data Sharing Statement

Kaltenhauser. Association of Body Mass Index and Waist Circumference With Imaging Metrics of Brain Integrity and Functional Connectivity in Children Aged 9 to 10 Years in the US, 2016-2018. *JAMA Netw Open*. Published May 18, 2023. doi:10.1001/jamanetworkopen.2023.14193

Data
Data available: No

Additional Information
Explanation for why data not available: All raw and processed data used in this study are obtained from the NIMH Data Archive (NDA) (https://nda.nih.gov/abcd) with the approval of the ABCD consortium. To obtain permission to these data, users must create an account through the NDA and follow the instructions on the website to gain access.
